# Molecular control of cellulosic fin morphogenesis in ascidians

**DOI:** 10.1186/s12915-024-01872-7

**Published:** 2024-04-02

**Authors:** Maxence Lanoizelet, Christel Elkhoury Youhanna, Agnès Roure, Sébastien Darras

**Affiliations:** 1 Sorbonne Université, CNRS, Biologie Intégrative Des Organismes Marins (BIOM), Banyuls/Mer, 66650 France; 2 Present address: Laboratory of Developmental Neurobiology, Department of Biology, KU Leuven, Louvain, Belgium; 3 Present address: Centre de Biologie Structurale, Univ Montpellier, CNRS UMR 5048, INSERM U1054, Montpellier, 34090 France

**Keywords:** Ascidian, Tunic, Cellulose, 3D morphogenesis, HGT, Median fin

## Abstract

**Background:**

The tunicates form a group of filter-feeding marine animals closely related to vertebrates. They share with them a number of features such as a notochord and a dorsal neural tube in the tadpole larvae of ascidians, one of the three groups that make tunicates. However, a number of typical chordate characters have been lost in different branches of tunicates, a diverse and fast-evolving phylum. Consequently, the tunic, a sort of exoskeleton made of extracellular material including cellulose secreted by the epidermis, is the unifying character defining the tunicate phylum. In the larva of ascidians, the tunic differentiates in the tail into a median fin (with dorsal and ventral extended blades) and a caudal fin.

**Results:**

Here we have performed experiments in the ascidian *Phallusia mammillata* to address the molecular control of tunic 3D morphogenesis. We have demonstrated that the tail epidermis medio-lateral patterning essential for peripheral nervous system specification also controls tunic elongation into fins. More specifically, when tail epidermis midline identity was abolished by BMP signaling inhibition, or CRISPR/Cas9 inactivation of the transcription factor coding genes *Msx* or *Klf1/2/4/17*, median fin did not form. We postulated that this genetic program should regulate effectors of tunic secretion. We thus analyzed the expression and regulation in different ascidian species of two genes acquired by horizontal gene transfer (HGT) from bacteria, *CesA* coding for a cellulose synthase and *Gh6* coding for a cellulase. We have uncovered an unexpected dynamic history of these genes in tunicates and high levels of variability in gene expression and regulation among ascidians. Although, in *Phallusia*, *Gh6* has a regionalized expression in the epidermis compatible with an involvement in fin elongation, our functional studies indicate a minor function during caudal fin formation only.

**Conclusions:**

Our study constitutes an important step in the study of the integration of HGT-acquired genes into developmental networks and a cellulose-based morphogenesis of extracellular material in animals.

**Supplementary Information:**

The online version contains supplementary material available at 10.1186/s12915-024-01872-7.

## Background

The tunicates are filter-feeder sea animals and are the closest invertebrate relatives of the vertebrates. They share with them a basic body plan most visible during embryonic life with the presence of a notochord and a dorsal hollow neural tube. Together with cephalochordates (amphioxus), vertebrates and tunicates form the chordates [1, 2]. A unique structure, the tunic, gives their name to the tunicates, unites them, and distinguishes them from other chordates. It consists of a “mantle” of extracellular material secreted by the epidermis that protects them from the outside world but also gives them their external appearance, shape, and color. It can be best compared to the insect exoskeleton, the cuticle. Tunic formation is poorly known and studied. However, a major and striking discovery was the identification of cellulose as a major component of the tunic. This biomolecule is the most abundant on earth, and it is best known as the primary compound of plant cell wall with multiple applications in human life (paper, textiles, chemistry…). Tunicates are actually the only metazoans known to synthesize cellulose, a specificity that appeared through the acquisition of a *cellulose synthase* (*CesA*) gene from a bacteria through horizontal gene transfer (HGT) [3–7]. This gene is expressed in the epidermis during embryogenesis and is essential for cellulose production and proper metamorphosis. Importantly, in the absence of *CesA*, a tunic still forms [7]. This is in agreement with the fact that cellulose is not the sole component of the tunic that includes other extracellular material such as polysaccharides and proteoglycans [8–12]. Very recently, another gene acquired through HGT, *Gh6*, has been described as expressed in the epidermis and possibly involved in cellulose metabolism and metamorphosis [13, 14].

The tunic as mentioned above gives their shape to the tunicates. The most striking example comes from a the appendicularians (or larvaceans). Their tunic, also called the “house,” has a sophisticated 3D structure with multiple parts and serves as a complex filtering and food—microalgae and bacteria—concentrating engine. Instead of cleaning the filter when clogged, appendicularians escape their house and build a new one, and they do so several times a day. This is an astonishing and fascinating case of 3D morphogenesis using extracellular matrix production that has been studied in some detail at the molecular level [6, 15–17]. We have chosen to turn to a much simpler case. Another group of tunicates, the ascidians (or sea squirts), possesses a tunic not only during the adult life but also during the larval life—a tadpole-like form. This larval tunic is longer along the dorsal, ventral, and caudal parts of the tail to form dorsal, ventral, and caudal fins. The median (dorsal and ventral) and caudal fins are thought to be essential for swimming [18]. In the trunk, the tunic also differentiates with some specific elongations [19]. We postulate that 3D morphogenesis of the larval ascidian tunic is regulated through specific secretion and/or modification of extracellular matrix components at the specific location where elongations/extensions occur. It is most likely that tunic shape is intimately linked to the patterning of the underlying epidermis. By studying the developmental mechanisms for the caudal peripheral nervous system (PNS) formation in the ascidian *Ciona intestinalis*, we have uncovered cellular and molecular patterning of the tail epidermis into three main longitudinal rows of cells: the dorsal and ventral midlines, the four medio-lateral rows, and the two lateral rows [20]. Interestingly, the larval tunic shape is similar in distantly related species (Additional file 1: Fig. S1) [21], and the tail epidermis transcriptional patterning and the underlying gene regulatory network (GRN) is partly conserved [22, 23].

Here, we have used the embryos of the ascidian *Phallusia mammillata* to interrogate the mechanisms of larval tunic morphogenesis. We showed that embryonic developmental mechanisms that pattern the tail epidermis also regulate tunic shape. Specifically, inhibition of BMP signaling that prevents ventral midline specification abolished ventral median fin formation. CRISPR/Cas9-mediated mutagenesis of *Msx* and *Klf1/2/4/17* coding for transcription factors expressed at the tail midlines impaired median fin elongation. We next sought for effectors of tunic elongation by focusing on HGT acquired cellulose-related genes, *CesA* and *Gh6*. We describe a dynamic evolutionary history of these genes in tunicates and their expression patterns and regulation in four ascidian species using cross-species transcriptional assays. Despite a shared epidermal expression and a conserved regulation of *CesA* by the epidermis determinant Tfap2, the expression and transcriptional regulation of these HGT genes is unexpectedly variable. Finally, we uncovered a conserved regionalized epidermal expression of *Gh6* that is regulated by the tail epidermis GRN. Functional studies indicated that *Gh6* is not a major regulator of fin formation but that the cellulase encoded by *Gh6* may participate in the restriction of tunic elongation during fin blade formation.

## Results

### Larval tunic formation in Phallusia mammillata

**Fig. 1:**
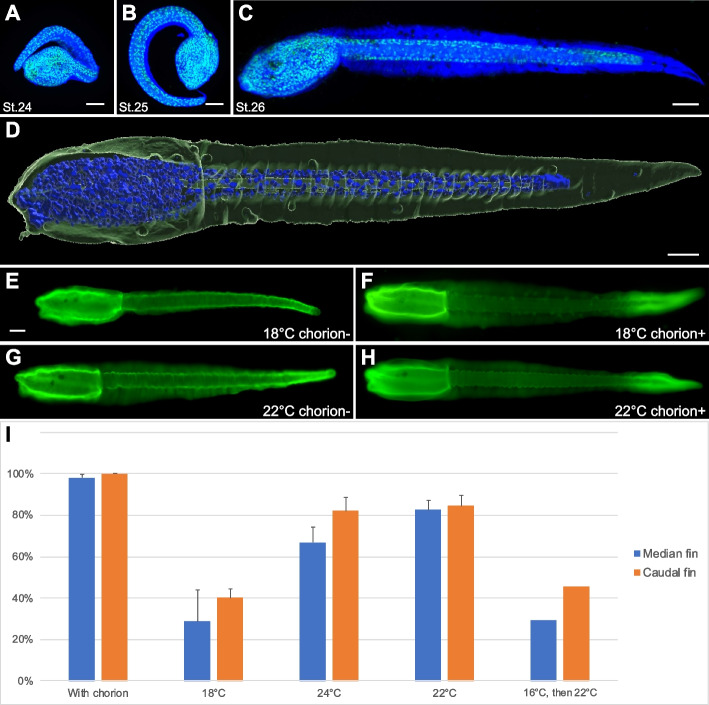
Tunic and fin blades development in *Phallusia mammillata*.** A–C** Embryos at St. 24 (**A**) and St. 25 (**B**) that developed in their chorions, and a hatching larva (**C**) were stained with calcofluor white (blue) and Sytox Green (green). Maximum intensity projections from confocal z-stacks (the corresponding 3D visualizations can be found in Additional file 3: Movie S1, Additional file 4: Movie S2 and Additional file 5: Movie S3). **D** 3D surface rendering from a confocal z-stack of a larva stained with CBM3a-GFP (green) and DAPI (blue) (the corresponding 3D visualization can be found as Additional file 6: Movie S4). **E–H** Effects of temperature during development on the formation of the tunic (stained with CBM3a-GFP in green) of larvae that arose from dechorionated eggs. Although the tunic of larvae that developed at 18 °C was devoid of median and caudal fins most of the time (**E**), the tunic of larvae that developed at 22 °C had more frequently median fins with a normal appearance and caudal fins with a reduced size (**G**). Chorionated embryos systematically gave rise to larvae with well-formed median and caudal fins (**F**,**H**). **I** Graph representing the proportions of larvae presenting median (blue) and caudal (orange) fins at the different culture temperatures (average values with error bars denoting standard deviation). With chorion: *n* = 78 (3 experiments: one at 16 °C, one at 18 °C, and one at 24 °C). 18 °C without chorion: *n* = 141 (3 independent experiments). 24 °C without chorion: *n* = 123 (3 independent experiments). 22 °C without chorion: *n* = 195 (4 independent experiments). 16 °C, then 22 °C without chorion: *n* = 48 from a single experiment. Individual data values can be found in Additional file 7: Table S1. Note that although the tunic was uniformly stained by CBM3a-GFP in dechorionated larvae, the tunic staining of chorionated larvae was always less intense in the middle part. Embryos in A and B have developed in their chorions, consequently they appear rolled up. Larvae in **C–H** are shown in lateral views with anterior to the left and dorsal to the top. Scale bars: 50 µm

Previous studies on the molecular control of larval tunic formation have been performed on *Ciona robusta* (*Ciona intestinalis* type A) [4, 7, 13]. We thus first described larval tunic formation during *P. mammillata* development. Since tunic is transparent, we have used three dyes, calcofluor white, DirectRed23, and CBM3a-GFP (see “Methods”) to visualize the tunic. Although all dyes may not be specific to cellulose since they might bind other polysaccharides [6], they were useful to follow the course of tunic formation (Fig. 1; Additional file 2: Fig. S2). Tunic was first seen at late tailbud stages (St. 24) as a thin layer surrounding the embryo (Fig. 1A). It progressively thickened with a small caudal fin visible at St. 25 (Fig. 1B). At hatching (St. 26), extended median and caudal fins were readily visible (Fig. 1C, D).

In order to manipulate ascidian embryos, the protective chorion is usually removed before or just after fertilization. It has been known for long time that embryos deriving from such treatment are not fully normal. In particular, left/right asymmetry and tunic formation are disrupted [21, 24, 25]. When we observed the tunic of larvae that derived from eggs whose chorion was removed before fertilization (using our routine protocol [26]), the tunic was stained using cellulose dyes, but median and caudal fins were extremely reduced in size, abnormal or absent (Fig. 1E, I) when compared with larvae that developed within their chorions (Fig. 1F, I). We fortuitously found that increasing the temperature of embryonic development partly compensated for the absence of chorion. Dechorionated larvae that resulted from a development at 22°C or 24°C possessed fins more frequently (this frequency being quite variable from batch to batch), albeit usually reduced in size (Fig. 1G, I). Interestingly, increasing the temperature before tunic and fin formation at early tailbud stages (St. 20/21) was not sufficient to recover fin formation (Fig. 1I). This observation suggests that early events (possibly gene regulation), rather than a biophysical effect of temperature on tunic production, are the targets of temperature.

### Tail epidermis patterning regulates median fin morphogenesis

**Fig. 2:**
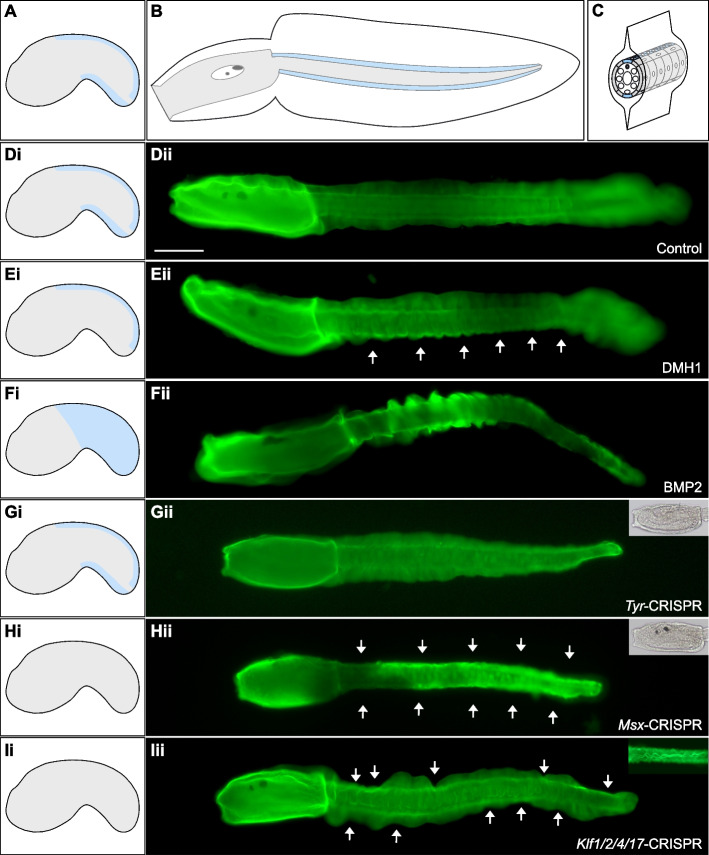
Molecular patterning of the tail epidermis regulates median fin formation. **A–C** Schematic representation of an early tailbud (**A**), a larva in lateral view (**B**), and a cross-section of the larval tail (**C**) with the embryo/larva itself in gray surrounded by the tunic. The tail epidermal neurogenic midlines that lie where median fins are positioned are highlighted in light blue. **D–F** Effects of BMP pathway modulation on median fin formation: control (BSA- and DMSO-treated larva) (**Dii**), DMH1-treated larva did not develop a ventral fin (**Eii**), and BMP2-treated larva with an excess of tunic making bulges (**Fii**). **G–I** Effects of CRISPR/Cas9 gene inactivation on tunic formation: *Tyr*-CRISPR larva formed a normal tunic with fins but lacked pigment cells (**Gii**), *Msx*-CRISPR larva lacked both ventral and dorsal median fins and had normal pigment cells (**Hii**), and *Klf1/2/4/17*-CRISPR larva presented indentations in the median fin that had an undulated shape (**Iii**). CBM3a-GFP staining of larvae in **D–I** (green). Insets in **Gii** and **Hii**: transmitted light picture of the trunk. Inset in **Iii**: dorsal view focused on the median fin. Larvae are shown in lateral view with anterior to the left and dorsal to the top. On the left panels (i): schematic representation of the distribution of the midline fate (light blue) in the different conditions. White arrows highlight the absence of median fin. Individual data values can be found in Additional file 7: Table S1. Scale bar: 100 µm

Median fin blades elongate above a specific epidermal cell population, the tail neurogenic midlines (Fig. 2A–C). We first inhibited the BMP signaling pathway that is necessary for ventral tail midline fate acquisition [20, 23, 27]. Strikingly, treated larvae had no ventral fin (DMH1: 71% of larvae with median fin had only a dorsal fin, *n* = 188; DMSO/BSA-control: 100% of larvae with median fin had both dorsal and ventral fins, *n* = 181; results from four independent experiments; Fig. 2D, E). A reciprocal experiment, treatment with recombinant BMP2 protein, leads to ectopic ventral midline fate in the entire tail epidermis [22, 23]. Contrary to our expectations, we did not observe a radial median fin all around the larval tail following such a treatment (Fig. 2F). A median fin was not recognizable anymore and the tunic was somehow inflated making bulges all around the tail (this phenotype was observed in 99% of the embryos, *n* = 272; results from three independent experiments). This observation is consistent with an excess of tunic production. We performed the same treatments on *C. intestinalis* embryos (Additional file 8: Fig. S3). Although DMH1 treatment also led to a loss of ventral median fin, BMP2 treatment was different: the median fin was readily visible but numerous isolated fibers were seen protruding out of the tunic. While we are not yet able to explain the differences in phenotype for both species, the results argue for an excess of tunic production following BMP pathway activation and epidermis midline fate expansion.

We next aimed at targeting downstream genes that belong to the tail PNS GRN using CRISPR/Cas9 gene editing. We used microinjection-mediated introduction of the ribonucleoprotein complex that has been recently described for *Phallusia* [28]. As a control, we targeted *Tyrosinase* that is essential for melanin pigmentation of sensory organs found in the larval brain, the otolith, and the ocellus. Absence of pigment cells was observed in 41% of larvae on average (59% with 2 pigment cells, 6% with 1 pigment cell, and 35% with no pigment cell; *n* = 101 from three independent experiments; Fig. 2G). We first examined the effects of mutating *Msx*, the gene coding for a homeodomain-containing transcription factor that lies at the top of the GRN [22, 29]. As anticipated, median fin formation was inhibited (*Tyr*-CRISPR: 79% of larvae with a median fin, *n* = 78; *Msx*-CRISPR: 35% of larvae with a median fin, *n* = 91; results from two independent experiments; Fig. 2H). The penetrance of this phenotype was not complete, but was in agreement with the fact that we observed that 6/10 larvae were mutated at the *Msx* locus in a separate experiment (Additional file 9: File S1). We then mutated *Klf1/2/4/17* that codes for a Zn-finger transcription factor acting downstream of *Msx* [29]. Surprisingly, we did not phenocopy *Msx*-CRISPR larvae. The frequency of fin formation was similar to control *Tyr*-CRISPR larvae (*Tyr*-CRISPR: 87% median fin, 43% caudal fin, *n* = 64; *Klf1/2/4/17*-CRISPR: 82% median fin, 46% caudal fin, *n* = 52; results of two independent experiments). However, median fins were strongly malformed with a seemingly random local reduction in size leading to a wavy edge of the median fins (F ig. 2I). This phenotype that was present in 77% of the larvae with median fins was never observed in *Tyr*-CRISPR, non-injected or non-dechorionated larvae. In addition, the median fin had an undulated shape, (Fig. 2Iii inset). Genotyping indicated that 7/8 larvae had a mutated *Klf1/2/4/17* locus in an independent experiment (Additional file 9: File S1).

The results of this section demonstrate that acquisition of midline fate in the epidermis is essential for median fin formation.

### Phylogenetic distribution, embryonic expression, and transcriptional regulation of cellulose-related HGT-acquired genes in ascidians

We hypothesized that elongation of the larval tunic into fin blades could be the result of specific regulation of cellulose production. We thus examined the phylogenetic distribution and embryonic expression of the two cellulose-related HGT-acquired genes *CesA* and *Gh6* in ascidians.

#### Pan epidermal expression of CesA

When searching for the ortholog of *CesA* in *P. mammillata* genome, we found two hits located on different scaffolds. Although predicted proteins differed in size, they had similar structures with seven transmembrane domains, a glycosyl transferase 2 (GT2)/cellulose synthase domain and a glycosyl hydrolase 6 (GH6)/cellulase domain (Additional file 10: Fig. S4). Both genes had similar temporal dynamics (expressed during late embryonic development), but their levels of expression were highly different, *CesA.b* being expressed at very low levels compared to *CesA*. To verify that the existence of these two genes was not an artifact of the *Phallusia* genome assembly, we surveyed tunicate genomic and transcriptomic data for *CesA* genes. As previously reported, we found a single *CesA* for most tunicates except *Oikopleura dioica* for which a lineage-specific duplication occurred [6, 30]. However, in addition to *P. mammillata*, we found three species that had 2 *CesA* genes (Additional file 11: Fig. S5; Additional file 17: Table S3): *Phallusia fumigata*, *Ascidia mentula*, and *Corella inflata*. Phylogenetic analysis demonstrated the existence of three clades (Additional file 11: Fig. S5): one that includes most previously described CesA, one with CesA proteins from *Oikopleura*, and one that includes the second copy present in these four species (we named these proteins CesA.b). The chromosomal assembly of *A. mentula* indicates that *CesA* and *CesA.b* are located on different chromosomes (Additional file 17: Table S3), suggesting that recent local tandem duplication is not the source of *CesA.b* emergence.

**Fig. 3:**
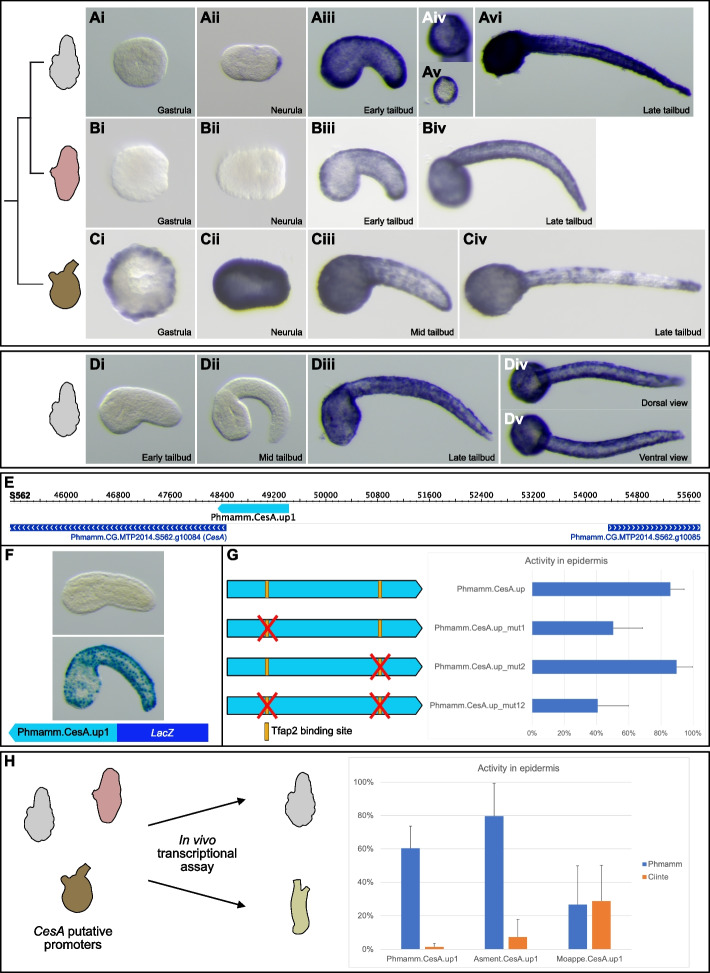
*CesA* expression and regulation in different ascidian species.** A–C** In situ hybridization for *CesA* during embryonic development of *Phallusia mammillata* (**A**), *Ascidia mentula* (**B**), and *Molgula appendiculata* (**C**). In *P.* mammillata, the expression was first detected in the caudal part of late neurulae (**Aii**). Expression was found throughout the epidermis with weaker staining in the palp forming region (**Aiv**). A cross-section of the tail shows that the staining was limited to the epidermis and not found in internal tissues (**Av**). A similar pattern was found in *A. mentula* (**B**) and *M. appendiculata* (**C**) but with a much earlier onset of expression in the latter species (early gastrula: **Ci**). **D** In situ hybridization for *CesA.b* during embryonic development of *P. mammillata*. Transcripts were found throughout the epidermis from late tailbud stages (St. 24) with weaker expression in dorsal and ventral tail midlines (**Diii–Dv**). Embryos at the following stages are shown: gastrula (St. 10–11) (**Ai**,**Bi**,**Ci**), neurula (St. 14–16) (**Aii**,**Bii**,**Cii**), early/mid tailbud (St. 20–22) (**Aiii-Av**,**Biii**,**Ciii**,**Di**,**Dii**), and late tailbud (St. 24–25) (**Avi**,**Biv**,**Civ**,**Diii-Dv**). **E–G** Transcriptional regulation of *Phmamm.CesA* in the epidermis. A region of around 1 kb, Phmamm.CesA.up1, situated immediately upstream of *CesA* (**E**) was PCR-amplified and placed upstream of the *LacZ* reporter in inverted orientation. When tested in *P. mammillata* embryos (**F**), it was inactive at early tailbud stages (St. 19/20, no embryo stained in the epidermis, *n* = 185, a single experiment) and active in the epidermis at late tailbud stages (St. 23/24, 60% of the embryos with epidermis staining, *n* = 703 from 3 independent experiments). This region contains 2 putative Tfap2 binding sites (yellow bars in **G**). A synthetic version of Phmamm.CesA.up1, Phmamm.CesA.up, and 3 variants with Tfap2 mutations were placed in “correct” orientation upstream of *LacZ*, and their activity tested at mid-tailbud stages (St. 22) is summarized on the graph on the right in G (Phmamm.CesA.up, *n* = 296; Phmamm.CesA.up_mut1, *n* = 282; Phmamm.CesA.up_mut2, *n* = 363; Phmamm.CesA.up_mut12, *n* = 268; results from three independent experiments). **H** Swap assay of putative *CesA* promoters from three ascidian species. Each region was tested in both *P. mammillata* (Phmamm, blue) and *C. intestinalis* (Ciinte, orange), and the activity at mid/late tailbud stages in epidermis is displayed on the graph on the right (Phmamm.CesA.up1: in Phmamm *n* = 703 from 3 experiments, in Ciinte *n* = 348 from 2 experiments; Asment.CesA.up1: in Phmamm *n* = 899 from 4 experiments, in Ciinte *n* = 896 from 3 experiments; Moappe.CesA.up1: in Phmamm *n* = 767 from 3 experiments, in Ciinte *n* = 712 from 3 experiments). All pictures of embryos are lateral views with dorsal to the top and anterior to the left, except: animal views (**Ai**,**Bi**), vegetal view (**Ci**), neural plate views (**Bii**,**Cii**) with anterior to the left, frontal view (**Aiv**), dorsal view (**Div**), ventral view (**Dv**), and cross-section of the tail (**Av**). Individual data values can be found in Additional file 7: Table S1

We next determined the expression of *CesA* in three species: *P. mammillata*, *A. mentula*, and *Molgula appendiculata* by in situ hybridization (Fig. 3A–C; Additional file 12: Fig. S6). For all species, *CesA* had a pan-epidermal expression during embryonic development like in *Ciona* [4, 5, 13]. However, in *M. appendiculata*, the expression was initiated much earlier (early gastrula stages) than in the other three species (late neurula stages). Despite *CesA.b* was expressed at much lower levels (Additional file 10: Fig. S4), we managed to detect its expression pattern during embryogenesis by in situ hybridization in *P. mammillata* (Fig. 3D; Additional file 12: Fig. S6). *CesA.b* was broadly expressed in the epidermis of late tailbud embryos (St. 24) with weaker expression in the tail ventral and dorsal midlines.

#### Shared and divergent mechanisms regulating CesA expression in the epidermis

We isolated Phmamm.CesA.up1, a 1-kb genomic fragment immediately upstream of *Phmamm.CesA*, that proves sufficient to drive epidermal activity (Fig. 3E, F). Since *Cirobu.CesA* is regulated by the transcription factor Tfap2-r.b [31], we searched for Tfap2 binding sites and identified two putative sites (Fig. 3G). Mutagenesis followed by in vivo transcriptional assay indicated that the proximal site was dispensable (this site was actually predicted only by the GCCN_3/4_GGC motif, and not by the Jaspar matrices) and the distal site participated in *CesA* expression (Fig. 3G). In fact, binding site mutation only diminished reporter activity whereas mutation of the single Tfap2 site fully abolished reporter activity in the *Ciona* experiment [31]. To further probe potential differences between *Ciona* and *Phallusia*, we tested the transcriptional activity of Phmamm.CesA.up1 in *C. intestinalis*. Surprisingly, it was inactive (Fig. 3H). We isolated Asment.CesA.up1, a 1.6-kb long putative promoter from *A. mentula* that contains three predicted Tfap2 binding sites. Similarly to Phmamm.CesA.up1, this region was active in *Phallusia* epidermis but not in *Ciona* epidermis (Fig. 3H). We next tested a candidate region from a distantly related species *M. appendiculata*. This 0.6-kb region containing two putative Tfap2 sites was equally active in both *Phallusia* and *Ciona* albeit at weaker levels than previous genomic fragments.

#### Patterned epidermal expression of Gh6

We had identified the presence, in tunicate genomes, of another HGT gene originating from bacteria that codes for a most likely functional cellulase containing a transmembrane domain and an extracellular glycosyl hydrolase family 6 domain [32] that we named *Gh6*. Before the present study was completed, this finding was published by colleagues [14]. Very recently, *Gh6* was described in *C. robusta* as being expressed in the epidermis during embryogenesis and as regulating larval tunic formation and metamorphosis [13]. We confirmed the published phylogeny of tunicate Gh6 proteins (Additional file 13: Fig. S7). We identified a single gene for each species, except for the Thaliacean *Salpa thompsoni* that presented a lineage-specific duplication (Additional file 13: Fig. S7) [14].

**Fig. 4:**
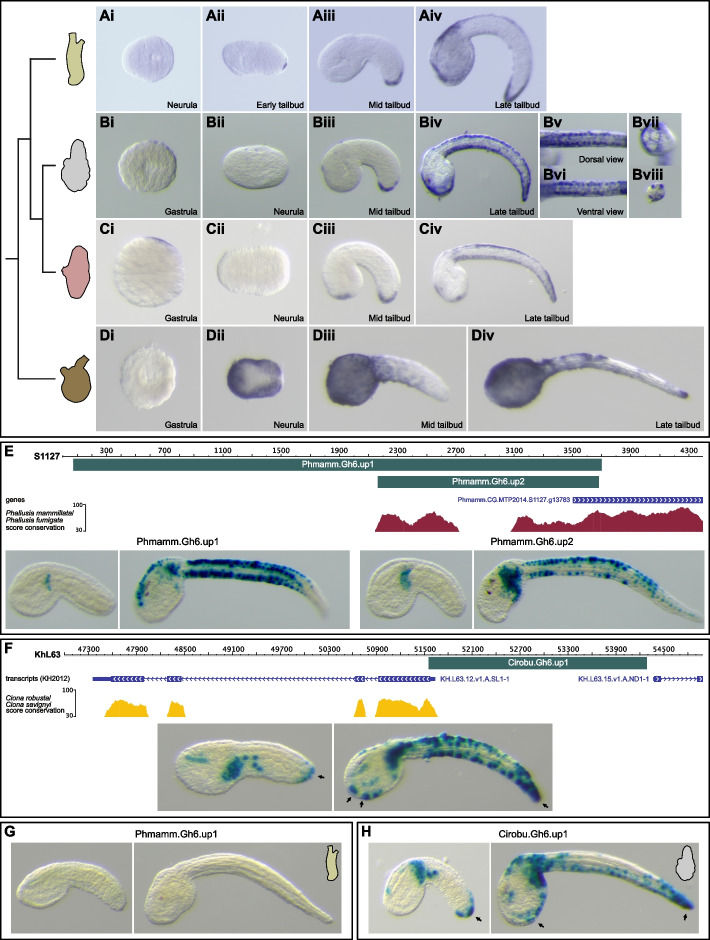
*Gh6* expression and regulation in different ascidian species.** A–D** In situ hybridization for *Gh6* during embryonic development of *Ciona intestinalis* (**A**), *Phallusia mammillata* (**B**), *Ascidia mentula* (**C**), and *Molgula appendiculata* (**D**). For the three phlebobranch species, *Gh6* was first detected at early tailbud stages in the epidermis at the tip of the tail. Then expression started anteriorly in the palp region and in the tail epidermis at late tailbud stages. Tail epidermis expression was detected in the four medio-lateral rows of cells (**Bv**,**Bvi**,**Bviii**). Expression in the palp region was likely absent from the future protruding papillae (**Bvii**). By contrast, *Moappe.Gh6* was expressed broadly in the epidermis starting at neurula stages (**Dii–Div**). Embryos at the following stages are shown: gastrula (St. 10–11) (**Bi**,**Ci**,**Di**), neurula (St. 14–16) (**Ai**,**Bii**,**Cii**,**Dii**), early/mid tailbud (St. 20–22) (**Aii**,**Aiii**,**Biii**,**Ciii**,**Diii**), and late tailbud (St. 24–25) (**Aiv**,**Biv–Bviii**,**Civ**,**Div**). **E** Transcriptional regulation of *Phmamm.Gh6* in the epidermis. A 3.6-kb region starting at the beginning of the scaffold 1127 immediately upstream of *Gh6*, Phmamm.Gh6.up1, was PCR-amplified, placed upstream of the *LacZ* reporter, and tested in vivo. It was active only in a part of the *Phmamm.Gh6* expression domain, the tail epidermal medio-lateral cells at late tailbud stages (St. 19–22: 3% of embryos with staining in this expression domain, *n* = 401; St. 24–25: 75% of embryos with staining in this expression domain, *n* = 521; results from four experiments). Phmamm.Gh6.up2, a 1.5-kb region embedded in Phmamm.Gh6.up1 containing conserved segments, had a similar activity (St. 19–22: 0% of embryos with this expression domain, *n* = 670 from three experiments; St. 24–25: 56% of embryos with this expression domain, *n* = 556 from four experiments). **F** Transcriptional regulation of *Cirobu.Gh6* in the epidermis. Cirobu.Gh6.up1, a 2.7-kb region that almost corresponds to the entire upstream intergenic region, fully recapitulated *Gh6* expression with tail tip activity (arrows) detected in early tailbuds, and palp (arrows) and tail epidermis activity in late tailbuds (St. 19–22: 45% of embryos with expression in endogenous territories, *n* = 300; St. 24–25: 73% of embryos with expression in endogenous territories, *n* = 300; results from two experiments). **G** Phmamm.Gh6.up1 was not active in *C. intestinalis* embryos (*n* = 367 from two experiments). **H** Cirobu.Gh6.up1 was active in *Gh6* expression domains in *P. mammillata* embryos (St. 21–22: 36% of embryos with expression in endogenous territories, *n* = 448 from three experiments; St. 23–24: 72% of embryos with expression in endogenous territories, *n* = 210 from two experiments). All pictures of embryos are lateral views with dorsal to the top and anterior to the left, except: animal views (**Bi**,**Ci**), vegetal view (**Di**), neural plate views (**Ai**,**Cii**,**Dii**) with anterior to the left, frontal view (**Bvii**), dorsal view (**Bv**), ventral view (**Bvi**), and cross-section of the tail (**Bviii**). Individual data values can be found in Additional file 7: Table S1

We examined the expression pattern of *Gh6* during the embryonic development of four ascidian species. As expected, *Ciinte.Gh6* was expressed like *Cirobu.Gh6* (Fig. 4A) [13] with first detectable expression in the epidermis of the tip of the tail at late neurula/early tailbud stages (Fig. 4Aii). Novel domains of expression in the epidermis appeared at late tailbud stages: palp region, dorsal epidermis at the trunk/tail junction, and at the dorsal and ventral aspects of the tail (Fig. 4Aiv). We found similar expression patterns in *P. mammillata* and *A. mentula* (Fig. 4B, C; Additional file 12: Fig. S6). In *Phallusia*, we identified tail expressing cells as the four medio-lateral longitudinal rows of cells (two ventral and two dorsal rows) [20] (Fig. 4Biv-Bvi, Bviii). In the palp region, *Gh6* transcripts were depleted from future papillary protrusions (Fig. 4Bvii). By contrast, *Moappe.Gh6* had a very different pattern (Fig. 4D). Although it was expressed in the epidermis, the expression was detected earlier (neurula stages) and very broadly (entire epidermis with a depletion from the posterior tail as development proceeds).

#### Shared and divergent mechanisms regulating Gh6 expression in the epidermis

To apprehend transcriptional regulation of *Gh6*, we isolated candidate *cis*-regulatory regions in both *Phallusia* and *Ciona*. In *Phallusia*, the largest region we could test (the *Gh6* locus lies at the edge of the currently available *P. mammillata* genome assembly MTP2014) reproduced late expression in the medio-lateral domains of the tail epidermis but not the palp and tail tip regions (Fig. 4E). A smaller fragment with DNA sequences conserved between *P. mammillata* and *P. fumigata* had a similar activity. In *Ciona*, the intergenic region upstream of *Gh6*, with no detectable DNA conservation between *C. robusta* and *C. savignyi*, recapitulated both the early tail tip expression and the later palp and medio-lateral tail epidermis expression (Fig. 4F). Surprisingly, although the regulatory elements isolated from *Phallusia* were inactive in *Ciona*, the elements from *Ciona* were active in *Phallusia* (Fig. 4G, H).

Altogether, the results from this section indicated an unexpected variability of *CesA* and *Gh6* at all level examined: phylogenetic distribution in tunicates, expression domains, and possibly expression regulation. Nevertheless, these HGT-acquired genes are clearly deployed in the epidermis during late embryogenesis.

### Gh6 regulates caudal fin formation

**Fig. 5:**
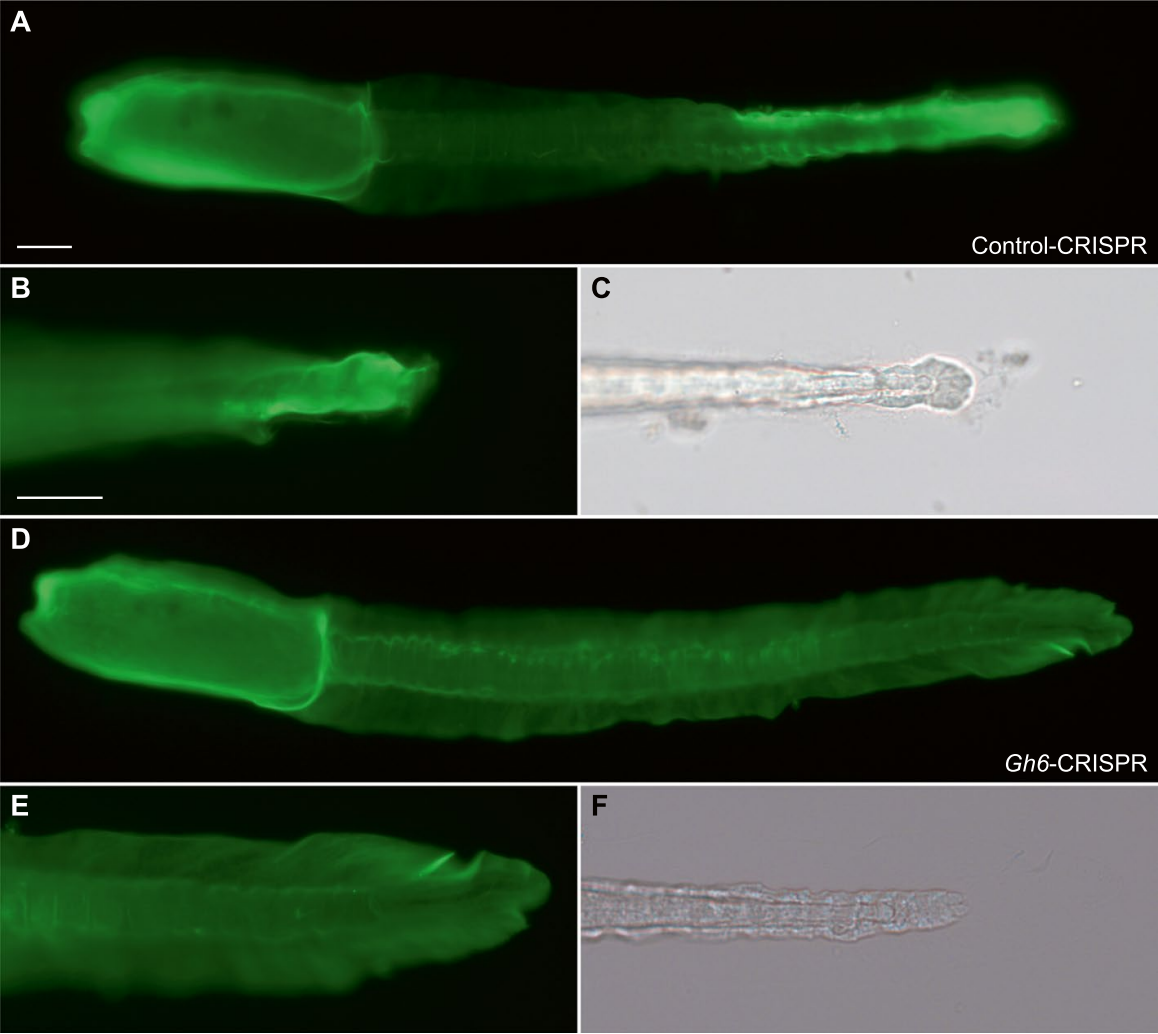
*Gh6* regulates caudal fin formation. Larvae resulting from microinjection of the CRISPR/Cas9 components targeting the *Brlanc.Ascl1/2.1* gene, a sequence absent from the *P. mammillata* genome (Control-CRISPR, **A–C**) or the *Phmamm.Gh6* gene (*Gh6*-CRISPR, **D–F**) were stained with CBM3a-GFP to visualize the tunic and fin blades. The caudal fin of the control larva ended where the tail ends (**B**,**C**). In contrast, the caudal fin of the *Gh6*-CRISPR larva was well developed. Scale bar: 50 µm. Individual data values can be found in Additional file 7: Table S1

As described previously (Fig. 2), modifying tail epidermis patterning impacted fin blade formation. Since *CesA.b* and *Gh6* displayed a patterned expression in the epidermis, we determined their expression following BMP pathway modulation (Additional file 14: Fig. S8). Their expression patterns were modified in agreement with their site of expression: loss of ventral medio-lateral *Gh6* expression and ectopic *CesA.b* expression at the ventral midline when BMP was inhibited. We thus functionally evaluated the role of *Gh6* in tunic and fin blade formation. We selected this gene for two reasons. First, *Gh6* is expressed closely to where median and caudal fins emerge (Fig. 4B). Second, by analogy with data from plants, we postulated that *Gh6* may contribute to fin blade elongation through local increase of cellulose production [33]. We first overexpressed Gh6 using pFog, an early pan-ectodermal driver that we have extensively used in *Ciona* and that is also active in *Phallusia* [20, 22, 23, 29]. We were surprised that median and caudal fins formed with the same frequency as in electroporation controls (Additional file 15: Fig. S9). We overexpressed Gh6 using two additional drivers (pSoxB2 active in the tail epidermis from the beginning of gastrulation [29] and Phmamm.CesA.up1 (Fig. 3)). We did not detect any effect on fin formation (Additional file 15: Fig. S9). To test the above model for *Gh6* function, we nevertheless turned to gene inactivation using CRISPR/Cas9 (Fig. 5). Contrary to our expectations, *Gh6* inactivation did not abolish fin elongation but rather promoted an opposite fin phenotype. While the median fin was present similarly in both control-CRISPR larvae (in 73% of larvae, *n* = 136 from two experiments) and *Gh6*-CRISPR larvae (69%, *n* = 155 from two experiments), the frequency of caudal fin presence increased from 48% in control-CRISPR larvae to 75% in *Gh6*-CRISPR larvae. Mutations of the *Gh6* locus by CRISPR/Cas9 were observed in 7/10 larvae that were genotyped independently (Additional file 9: File S1). Overall, our functional analysis indicates that *Gh6* is not the major effector of fin morphogenesis downstream of the epidermis patterning developmental network or that it plays a minor role possibly in conjunction with other yet unidentified actors.

## Discussion

Using functional approaches in *Phallusia*, we have shown that in the tail epidermis, midline fate acquisition through the gene network regulating PNS formation is essential for larval fin blade formation. By studying candidate HGT-genes *CesA*, *CesA.b,* and *Gh6* coding for enzymes regulating cellulose metabolism, we uncovered unanticipated diversity in gene expression and regulation. Finally, despite modest phenotypes, experiments of gain- and loss-of-function for *Gh6* suggest that it may act as a negative regulator of fin formation.

### Function of Gh6 in tunic formation?

**Fig. 6:**
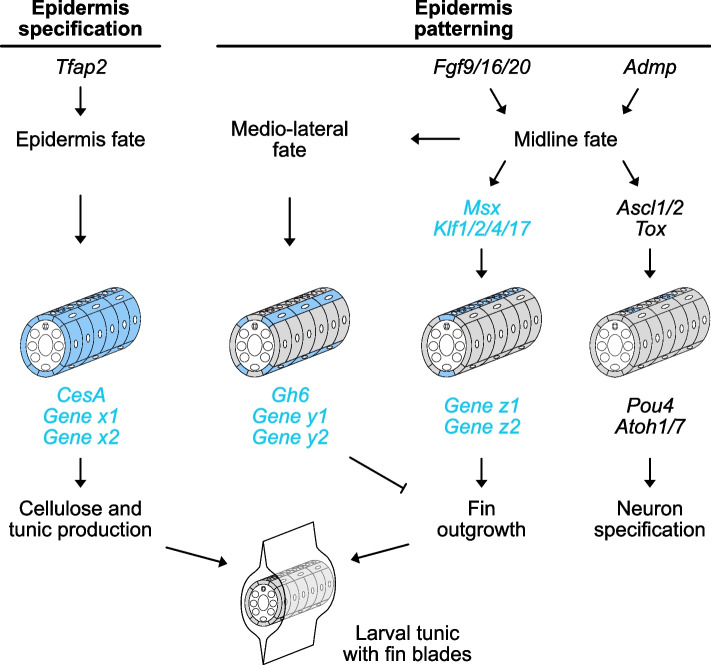
Summary model for the molecular control of median fin formation. Epidermis specification by *Tfap2* regulates the HGT-gene *CesA* and unidentified genes (*Gene x*) whose products are responsible for synthesis and secretion of cellulose and other components of the tunic in all epidermal cells. Epidermis patterning initially launched by *Fgf9/16/20* (dorsal midline induction) and *Admp* (ventral midline induction) activates two separate networks: one that regulates peripheral sensory neuron specification, and one that regulates fin outgrowth. Although *Msx* and *Klf1/2/4/17* are essential for fin formation, their downstream effector(s) are not known (*Gene z*). Medio-lateral fate is most likely regulated by midline cells (for example, in DMH1-treated embryos, midline fate is lost and *Gh6* fails to get expressed in the ventral medio-lateral cells, see Additional file 14: Fig. S8). Medio-lateral cells express the HGT-gene *Gh6* and possibly other genes (*Gene y*) that inhibit fin outgrowth

Cellulases have an obvious function of cellulose degradation during digestion for example. However, in both bacteria and plants, cellulases are part of the cellulose production machinery and promote the formation of crystalline over amorphous cellulose [33, 34]. For example, in *Arabidopsis*, KORRIGAN are cellulase mutants that present reduced biomass production. Although the enzymatic domains are different (GH8 in bacteria, GH9 in plants, and GH6 in tunicates), we postulated an analogous function for *Gh6* that would participate in local cellulose increased production and fin blade elongation. Instead of a reduction of fin blade elongation, we observed “better” caudal fin formation in our CRISPR experiment (Fig. 5). This suggests that *Gh6* normally prevents/regulates excessive fin elongation that would be triggered by another mechanism (Fig. 6). However, the lack of effect of Gh6 overexpression (Additional file 15: Fig. S9) questions its actual enzymatic activity. But it is worth keeping in mind that *Gh6* is predicted to encode a cellulase with a transmembrane domain and a GH6 domain facing the extracellular space. Hence, during overexpression, Gh6 that does not diffuse throughout the extracellular matrix might not be able to elicit a phenotype in the tunic. Biochemistry experiments would thus be needed to determine whether Gh6 acts as a glycoside hydrolase and whether it does so on cellulose or other carbohydrates.

In a recent study, TALEN-mediated *Gh6* mutagenesis in *Ciona robusta* has also ascribed a function for *Gh6* in tunic formation with an excess of tunic where sensory papillae normally protrude [13]. In addition, the formation of these papillae was prevented. This is an interesting phenotype given the regionalized expression of *Gh6* at this location (Fig. 4). In our experiments, we did not observe any phenotype in the palps. The difference could stem from species-specific functions of *Gh6*, but the specificity of the reported phenotype in *Ciona* is questioned by the authors themselves since it could stem from toxicity of the TALEN system [13]. It is also important to note that fin formation has not been examined in the cited study.

We were surprised by the modest phenotype for *Gh6*-CRISPR. A hint at the non-essential role of *Gh6* in fin formation comes from our description of its expression in different species (Fig. 4). In *Molgula*, *Gh6* was found epidermal but without regionalized expression. It is thus unlikely to regulate local tunic expansion into fin blades. While this observation could stem from an inherent divergence in this species which belongs to a very fast-evolving genus, a better candidate would be a gene with conserved regionalized expression in all ascidians that make fin blades (Additional file 1: Fig. S1). Tunicate genomes encode a number of glycosyl hydrolases (annotated for *Phallusia* in the CAZY database [32]), and one of them might fulfill the requirements for our above model for fin elongation. We are currently searching for genes coding for cellulase expressed in the epidermis during late embryogenesis in different ascidian species.

### Cellulose-related HGT-acquired genes in ascidians: dynamic evolutionary history and developmental system drift.

HGT-based capacity of producing cellulose is at the core of tunic formation, a defining feature of the tunicate phylum. As discussed in previous reports [4, 6, 14, 30, 31], the process is not limited to the initial ancient cross-kingdom gene transfer. Beyond the open question of the number of HGT events (Have *CesA* and *Gh6* been acquired simultaneously? What about other yet to be described HGT-acquired genes?), such events imply novel functionalities for the host, hence maintenance of the gene and control of its expression. We discuss the integration of *CesA* and *Gh6* into developmental GRNs in the next section. Here, we would like to emphasize the surprisingly elevated degree of variations we have uncovered at different levels: evolutionary history, gene expression, and gene regulation, despite very similar tunic and fins in ascidian tadpole larvae. We have discovered a novel lineage-specific duplication of *CesA* in the ascidian sub-lineage comprising *Phallusia* and *Ascidia*. A similar case has been described in the appendicularian *Oikopleura dioica* where the two paralogs are deployed during separate parts of the life cycle and in different body parts (*CesA1* during the embryonic phase in the lateral tail epidermis and *CesA2* during the adult phase in the trunk epidermis). Since we have no data about gene expression outside the embryonic period in *Phallusia*, we are not able to tell whether we are in the presence of a similar scenario. We could nevertheless describe co-expression of *CesA* and its paralog *CesA.b* in the epidermis of late embryos. The functional relevance of this observation and the regionalized expression of *CesA.b* will await further experiments. We have observed similar expression patterns for *CesA* and *Gh6* expression in the three phlebobranch species, but striking differences for *Molgula*, a stolidobranch ascidian (Figs. 3 and 4). This could be, as discussed above and earlier [23], that *Molgula* is an outlier. This will require further documentation on gene expression at broader scale in ascidians. Nevertheless, this is very suggestive that even phylum-defining components are subject to drift and are very plastic. More surprisingly, we uncovered possible differences in transcriptional regulation for both genes between *Ciona* and *Phallusia*: promoters of *Phallusia* were not active in *Ciona*, whereas the *Gh6* promoter of *Ciona* was active in *Phallusia*. We cannot formally conclude that the regulatory mechanisms have changed between *Ciona* and *Phallusia* without identifying the upstream transcription factors and their binding sites. However, this *“asymmetric intelligibility”* is a situation that we have not observed when we analyzed a number of developmental regulators [23], but that has been reported between *Ciona* and *Molgula*, species that have separated a longer time ago [35].

### Integration of the tunic production machinery to the developmental genetic programs

By studying tail PNS formation, we had uncovered medio-lateral cellular and molecular epidermis patterning [20]. Here, we show that the early inducing cue BMP signaling and downstream nodes of the GRN, *Msx*, and *Klf1/2/4/17* also regulate median fin formation (Fig. 2). It would thus be important to understand how HGT-acquired genes or yet unidentified effectors of fin elongation are integrated into the tail PNS GRN by dissecting the *cis*-regulatory regions of genes such as *Gh6*. Importantly, the tail PNS GRN that we and others have been deciphering [20, 22, 23, 29, 36–39] most likely diverges at some point into a “neuronal GRN” and a “fin blade GRN” since the tail midlines give rise to both peripheral sensory neurons and fins (Fig. 6). Comparative work among ascidians should help pointing out these sub-networks. We have identified divergent expression patterns for nodes of the PNS GRN [22, 23]: some genes were expressed throughout the midlines in all species examined whereas some genes were expressed only in a sub-domain in some species. It turned out that in these latter species, peripheral neurons do not form all along the midlines but only in parts of them. This is thus most likely that “pan-midline” genes fit into the “fin blade GRN” (all ascidian larvae have a median fin) and that genes with divergent expression fit into the “neuronal GRN”. This will be an exciting line of comparative functional exploration.

### Cellulose, extracellular material, and fin blade 3D morphogenesis

The molecular control of tunic and fin formation is yet underexplored. There are a number of open questions arising from previous studies and the present manuscript (Fig. 6). Cellulose is an essential emblematic component of the tunic. However, biochemical studies indicate that other polysaccharides and extracellular matrix components, in particular glycoproteins, make up the tunic. Moreover, *CesA* inactivation clearly shows that without cellulose, a tunic still forms but with an abnormal fin [7]. In addition, *CesA* alone is unlikely sufficient for cellulose synthesis since a number of molecular partners are required [4].

The larval cellulosic fins are essential for swimming. Consequently, they are likely essential for a key life cycle transition: the larval dispersal and most importantly the settlement before metamorphosis and the switch to a sessile form. Re-investigating tunic composition through the angle of molecular EvoDevo sounds a promising approach to understand extracellular tunic 3D morphogenesis into fin blades by combining developmental GRN data with scRNA-seq data and CRISPR/Cas9 gene inactivation studies in several ascidian species with sequenced and annotated genomes.

## Conclusions

Our study has demonstrated that acquisition of midline identity in the tail epidermis through developmental gene networks is responsible for the morphogenesis of the tunic extracellular matrix into fin blades. We have identified the HGT-gene *Gh6* as a negative regulator of fin formation, but the effector(s) promoting fin outgrowth have yet to be identified.

## Methods

### Embryo obtention and manipulation

Adults from *Ciona intestinalis* (formerly referred to *Ciona intestinalis* type B [40]) and *Ascidia mentula* were provided by the Centre de Ressources Biologiques Marines in Roscoff (EMBRC-France). *Phallusia mammillata* and *Molgula appendiculata* were provided by the Centre de Ressources Biologiques Marines in Banyuls-sur-mer (EMBRC-France). Gamete collection, embryo rearing, and electroporation were performed as described previously [23, 26]. Staging of embryos is according to the developmental table of *Ciona robusta* [41, 42]. To improve tunic and fin blades formation after dechorionation, embryos were cultured at 22°C (*P. mammillata*) or 19°C (*C. intestinalis*) in the presence of 0.1% BSA on 1% agarose-coated dishes. Dechorionated embryos were treated with 2.5 µM of the BMP receptor inhibitor DMH1 and with 150 ng/ml of recombinant BMP2 protein from early gastrula stages (St.10) as previously described [22, 27]. In vivo transcriptional assays were performed as described [23, 26].

Gh6 overexpression was performed by introducing, via electroporation into fertilized eggs, constructs containing the *Gh6* coding sequence under the control of various promoters: pFog (entire ectoderm from the 16-cell stage) [43], pSoxB2 (tail ectoderm from the 64-cell stage) [29], and pCesA (entire epidermis from early tailbud stages, Fig. 3). Electroporation was performed as previously described [26] with 50 µg plasmid DNA and using pFog > LacZ [43] as a control.

### Cellulosic larval fin visualization

When *P. mammillata* embryos reached the early swimming larval stage (St. 27; 16 hpf at 22°C), we transferred them in 2-ml low-binding microtubes in 1 ml of sea water. They were fixed for 2 h at room temperature (RT) or overnight (O/N) at 4°C by adding 1 ml of fixation buffer (0.1 M MOPS, 0.5 M NaCl, 7.4% formaldehyde) to reach a final concentration of 3.7% of formaldehyde. After three successive washes in PBST (137 mM NaCl, 10 mM Na_2_HPO_4_, 2.68 mM KCl, 1.76 mM KH_2_PO_4_, 0.1% Tween20), we proceeded to the cellulose staining step. Three different methods were optimized: (1) 2 h at RT or O/N at 4°C in 75 µg/ml of CBM3a-GFP (CZ00571, Nzytech), (2) 1 h at RT in 0.1% calcofluor white (#910090, Sigma-Aldrich), and (3) 3 h at RT in 0.1% Direct Red 23 (#212490, Sigma-Aldrich) in PBST. Staining was stopped by three successive washes in PBST. Nuclei were stained with 0.5 μg/ml DAPI (#62247, Fischer Scientific) or with 1 μM Sytox Green (S7020, Invitrogen) in PBST for 30 min at RT. CBM3a-GFP cellulose staining has been preferred since it did not produce non-specific staining of the larval body. Whole larvae were examined under a stereomicroscope (Olympus SZX16 equipped with a X-Cite Fluorescence Lamp Illuminators (Excelitas Technologies)), and imaged with a widefield inverted fluorescence microscope (Olympus model IX51HBO 100W equipped with a FL20 camera, Tucsen), and/or with a TCS SP8 X confocal microscope (Leica). 3D reconstruction and object modeling were conducted with the IMARIS microscopy imaging software (Oxford Instrument).

### Molecular biology, gene identifiers, and phylogeny

Gene identifiers, sequences and methods for RNA probe DNA templates and *cis*-regulatory DNA generation are described in Additional file 16: Table S2. Genes and *cis*-regulatory regions were named according to the ascidian community nomenclature [44].

Putative Tfap2 binding sites were mapped on *CesA* loci using FIMO (http://meme-suite.org/tools/fimo) [45] with matrices collected from the Jaspar database [46] and the GCCN_3/4_GGC motif [47]. The sites were mutated by making the following changes G- > C and C- > G in order to keep the GC content unchanged (the validity of these changes was confirmed using FIMO). Wild-type and mutated versions of the *Phmamm.CesA* promoter were synthesized as gBlocks (IDT).

Genomic regions that were amplified from genomic DNA using PCR or gBlocks were placed upstream of the *Ciinte*.*Fog* basal promoter and *LacZ* using the Gateway technology (Invitrogen) [23, 48].

Protein sequences for CesA and Gh6 were collected using blast from different genomic and transcriptomic resources listed in Additional file 17: Table S3. All sequences were aligned using the MUSCLE program [49]. Maximum-likelihood phylogenies were inferred using IQ-TREE (http://iqtree.cibiv.univie.ac.at/) [50].

Constructs for Gh6 overexpression. pFog > Gh6 and pSoxB2 > Gh6 were generated using the Gateway technology with a pENTRY clone containing the *Gh6* coding sequence (initially generated by PCR from a cDNA clone (Additional file 16: Table S2) with CAGAAAAA ATG GCGATGTCAAAGCGATG and TCA CCTGTTGATGCCAAACG) and the pDEST containing the promoters [29, 43, 48]. pCesA > Gh6 was generated by In-Fusion cloning (Takara) between one fragment containing the *Gh6* coding sequence (amplified by PCR from a cDNA clone (Additional file 16: Table S2) with GTACCGAGCT CAGAAAAA ATG GCGATGTCAAAGCGATGT and TGGCCTGCCCGGTTATTA TCA CCTGTTGATGCCAAACGG) and one fragment containing the *CesA* promoter and the pSP1.72 backbone (amplified by PCR from pSP1.72-Phmamm.CesA.up1-bpFOG-nlsLacZ (Additional file 16: Table S2) with TAATAACCGGGCAGGCCATG and TTTTTCTG AGCTCGGTACCCT).

### In situ hybridization

Chromogenic in situ hybridization in the different ascidian species were performed using the protocol described earlier [23, 27] with dig-labeled RNA probes described in Additional file 16: Table S2.

### CRISPR/Cas9 gene inactivation

The procedure was adapted from a recent protocol based on microinjection of the RNP complex into *Phallusia* [28]. Target sequences were designed with the help of CRISPOR (http://crispor.tefor.net/) [51]. We selected sequences with a high “Doench score” that target specific parts of the locus: one against the region coding for the N-terminal region of the protein and two against the region coding for functional domains. The sequences are shown in Additional file 18: Table S4. *P. mammillata* unfertilized eggs were dechorionated [26] and injected with the following solution: 30 μM pgRNA (duplex between custom sequence-specific crRNA and universal tracrRNA (IDT), 18.6 μM Cas9 (#1081058, IDT), 9 mM Hepes, 67.5 mM KCl, 2.2 mM CHAPS, 1.3% Fast Green, and 0.25% AlexaFluor555-dextran (D34679, Invitrogen) in nuclease-free Duplex Buffer (IDT). For control embryos, a single pgRNA (against *Phmamm.Tyr* or *Brlanc.Ascl1/2.1* depending on the experiment) at 30 µM was used. For *Msx*, *Klf1/2/4/17,* and *Gh6*, an equimolar mix of 3 pgRNAs totaling 30 µM was used. Injection was performed with a Femtojet micro-injector (Eppendorf) using needles pulled with a P-1000 Micropipette Puller (Sutter Instrument) starting with capillaries (0.1 OD × 0.78 ID × 100 L mm, GC100TF-10, Harvard Apparatus). One to three hours after injection, eggs were fertilized and left to develop at 22°C. Properly injected larvae were sorted using the AlexaFluor555-dextran fluorescence under a dissecting scope. Given the variability in tunic and fin formation from dechorionated embryos, a set of larvae injected with the control pgRNA was systematically generated and analyzed on the same batch of eggs as for the experimental pgRNAs targeting the gene of choice.

### Genotyping of CRISPR larvae

Individual larvae were collected in 20 µl of QuickExtract™ DNA Extraction Solution (Lucigen Corporation), and their genomic DNA was extracted according to the provider’s protocol. The targeted loci were amplified using a single PCR for *Msx* (Fwd: AGCCCCATCCCGAATTGACA, Rev: ATGATGCCCACTCGTCCACA) or using two-step nested PCRs for *Klf1/2/4/17* (PCR1: Fwd: ACCGCGAAATTGAGGCGTTT, Rev: CCACAGCGTTCGGAAACACA; PCR2: Fwd: AATGGTAAGGTGTTCGCCGC, Rev: CACACAGGGTTCTTGGCACC) and *Gh6* (PCR1: Fwd: GCGCTGTCTGTTACCTGGGA, Rev: CGCAGACGCCAAGAGAAGTG; PCR2: Fwd: GCTGTGCGAGCAGGTTTTGA, Rev: GGCAACCAAACCTCAGGCAC). Amplicons were loaded on agarose gels and Sanger sequenced from both ends with the PCR primers (Additional file 9: File S1).

### Supplementary Information

**Additional file 1: Fig. S1.** An ascidian zoo of cellulosic larval tunics. Larvae of various species, whose name is depicted below the image, were stained with DAPI (blue) and CBM3a-GFP (green). Median and caudal fins are clearly visible for all species. The maximum intensity projections are placed along a phylogenetic tree based on recent studies [52, 53].

**Additional file 2: Fig. S2.** Tunic staining using DirecRed23 and Calcofluor White. Both dyes stained the embryos quite strongly, but they were useful in delineating the tunic. (Top panel) DirectRed23 and DAPI staining. The first picture shows an overlay of DirectRed23 and DAPI, and is not informative. However, masking the embryo's shape using the DAPI channel (thanks to the background staining of the entire cell and not only the nucleus) allows a clear visualization of the tunic. Results on the presence of the tunic before hatching are similar to the ones obtained with Calcofluor White (Fig. 1 and below). (Bottom panel) Calcofluor White and Sytox Green staining. Similar results were obtained using a similar approach with the confocal acquisitions shown in Fig. 1A-C. All images are maximum intensity projection from confocal z-stacks.

**Additional file 3: Movie S1.** Animated maximum intensity projection of a z-stack of a St. 24 embryo stained with Calcofluor and Sytox Green.

**Additional file 4: Movie S2.** Animated maximum intensity projection of a z-stack of a St. 25 embryo stained with Calcofluor and Sytox Green.

**Additional file 5: Movie S3.** Animated maximum intensity projection of a z-stack of a St. 26 larva stained with Calcofluor and Sytox Green.

**Additional file 6: Movie S4.** 3D rotation of surface rendering from a z-stack of a larva stained with CBM3a-GFP and DAPI.

**Additional file 7: Table S1.** Individual data values.

**Additional file 8: Fig. S3.** Effects of BMP pathway modulation on larval tunic formation. (A-F) Phallusia mammillata. Overlay pictures between CBM3a-GFP and transmitted light for larvae from similar experiments as the ones described in Fig. 2 in lateral view (A,C,E) and dorsal views (B,D,F).  (G-I) Ciona intestinalis. Dechorionated embryos were treated with DMSO and BSA (control, G, *n*=57), 2.5 µM DMH1 (H), or 150 ng/ml recombinant BMP2 protein (I) from early gastrula stages (St. 10). The resulting larvae were stained with CBM3a-GFP (green). Note the absence of ventral fin in DMH1-treated larva (white arrows; observed in all of the 87 larvae examined). The median fin of BMP2-treated larva appeared normal, but numerous fibers protruding outside the tunic were visible (inset in I, observed in 96% of the 55 larvae examined). Results from a single experiment. Scale bar: 100 µm. Individual data values can be found in Additional file 7: Table S1.

**Additional file 9: File S1.** Evaluation of the mutagenesis triggered by CRISPR/Cas9. For each gene, a figure depicts: (top panel) the locus with the gene structure, essential protein domains, and the positions of sgRNA targets and PCR primers; (middle panel) a picture of an agarose gel for the different amplicons; and (bottom panel) results of Sanger sequencing at sgRNA targets' positions. Larvae with mutated locus are highlighted in orange. Each of these figures corresponds to a single experiment.

**Additional file 10: Fig. S4.** Phmamm.CesA and Phmamm.CesA.b genes: predicted proteins and expression during embryogenesis. Identified protein domains using NCBI conserved domain and EMBL-EBI HMMER search engines [54, 55] are highlighted with colors. Expression levels across embryonic stages based on RNA-seq data were retrieved from the Aniseed database [56].

**Additional file 11: Fig. S5.** Phylogenetic tree of CesA proteins in selected tunicate species. The tree was calculated using maximum likelihood (ML) method with IQ-TREE, and bootstrap supports are given at each node. Different groups are highlighted with colors: CesA (blue), CesA.b (purple), and CesA from Oikopleura (orange). The genes/proteins that are presented in this study are shown in red.

**Additional file 12: Fig. S6.** Additional images of in situ hybridization for CesA, CesA.b and Gh6 in Phallusia mammillata. In the top part of the figure, for each gene, the same embryo has been imaged through different orientations. In the bottom part of the figure, some pictures already presented in Figs. 3 and 4 are shown for clarity, in particular cross-sections through the tail are twice bigger than other pictures. Schematic expression domains are highlighted in blue. Note that these schemes do not account for the tail tip region where medio-lateral rows of cells most likely are missing. In the tail tip, we suspect that CesA.b is expressed in the lateral cells and Gh6 in the median cells. Scale bar: 100 µm (except cross-sections: 50 µm).

**Additional file 13: Fig. S7.** Phylogenetic tree of Gh6 proteins in selected tunicate species. The tree was calculated using maximum likelihood (ML) method with IQ-TREE, and bootstrap supports are given at each node. Different taxonomic groups are highlighted with colors: ascidians (blue), thaliaceans (purple), and appendicularians (black). The genes/proteins that are presented in this study are shown in red.

**Additional file 14: Fig. S8.** HGT gene expression regulation by BMP signaling pathway in Phallusia mammillata. In situ hybridization at late tailbud stages (St. 24/25) for CesA (A,D,G), CesA.b (B,E,H) and Gh6 (C,F,I) in control (A-C), DMH1-treated (D-F) and BMP2-treated (G-I) embryos. CesA expression was expressed in the entire epidermis in all conditions. DMH1 treatment led to a loss of Gh6 expression in the ventral epidermis medio-lateral rows of cells (black arrows), and to an increased expression of CesA.b in the ventral tail epidermis midline. BMP2 treatment led to disorganized 'salt-and-pepper' pattern for Gh6 and to a loss of CesA.b expression. The results come from two experiments for CesA and Gh6, and three experiments for CesA.b (the averaged fraction of embryos displaying the phenotype and the number of embryos is shown on each panel). Individual data values can be found in Additional file 7: Table S1. Embryos are shown in lateral views with dorsal to the top and anterior to the left except for CesA.b that are ventral views with anterior to the left. For each image, a schematic cross-section through the tail depicts our interpretation of the patterns. Scale bar: 100 µm.

**Additional file 15: Fig. S9.** Gh6 overexpression does not affect larval fin formation. Graphs representing scoring of median fin (top) and caudal fin (bottom) formation (following CBM3a-GFP staining) in larvae electroporated with the construct indicated on the graphs. The results come from 3 independent experiments with the following number of larvae examined: pFog>LacZ (234), pFog>Gh6 (148), pSoxB2>Gh6 (228), and pCesA>Gh6 (121). The error bars represent standard deviations. Individual data values can be found in Additional file 7: Table S1.

**Additional file 16: Table S2.** Identifiers and sequences for molecular biology (probe templates and cis-regulatory regions).

**Additional file 17: Table S3.** List of gene identifiers.

**Additional file 18: Table S4.** List of target sequences for CRISPR/Cas9.

## Data Availability

All data generated or analyzed during this study are included in this published article and its supplementary information files.

## Abbreviations

HGT: Horizontal gene transfer
PNS: Peripheral nervous system
GRN: Gene regulatory network
BMP: Bone morphogenetic protein
GH: Glycosyl hydrolase
GT: Glycosyl transferase
