# Supplementary figures and images for: Molecular control of cellulosic fin morphogenesis in ascidians
Source: BMC Biol. 2024 Apr 2;22:74. doi: 10.1186/s12915-024-01872-7 (PMC10986139; doi:10.1186/s12915-024-01872-7)

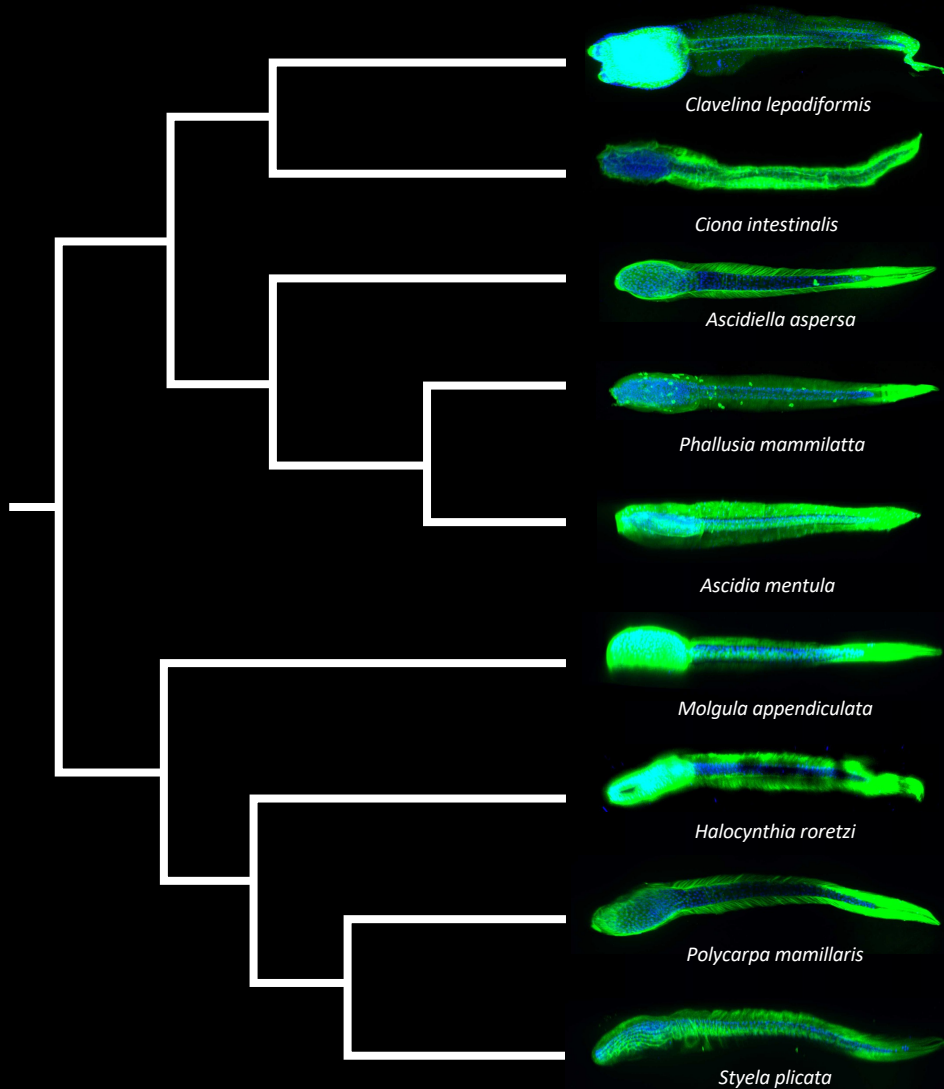

Aplousobranchia

Phlebobranchia

Stolidobranchia

Supplement: Supplementary file 1 — Additional file 1: Fig. S1. An ascidian zoo of cellulosic larval tunics. Larvae of various species, whose name is depicted below the image, were stained with DAPI (blue) and CBM3a-GFP (green). Median and caudal fins are clearly visible for all species. The maximum intensity projections are placed along a phylogenetic tree based on recent studies [52, 53]. [file 12915_2024_1872_MOESM1_ESM.pdf]

Lateral view

Dorsal view

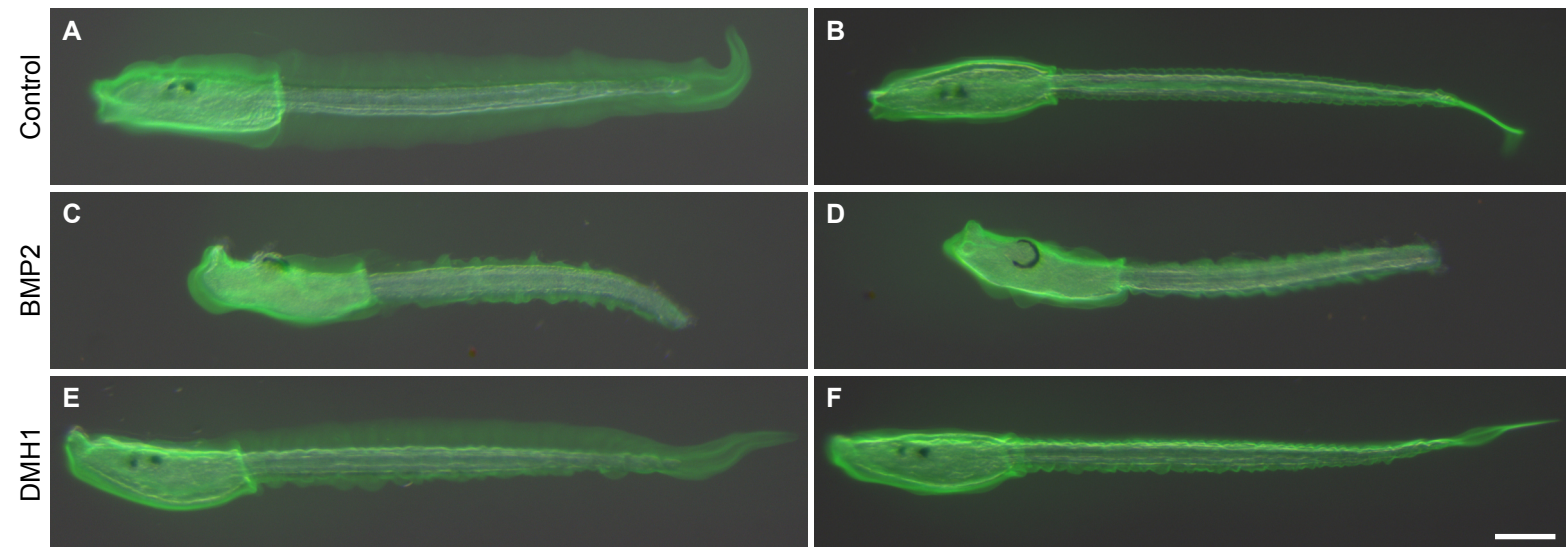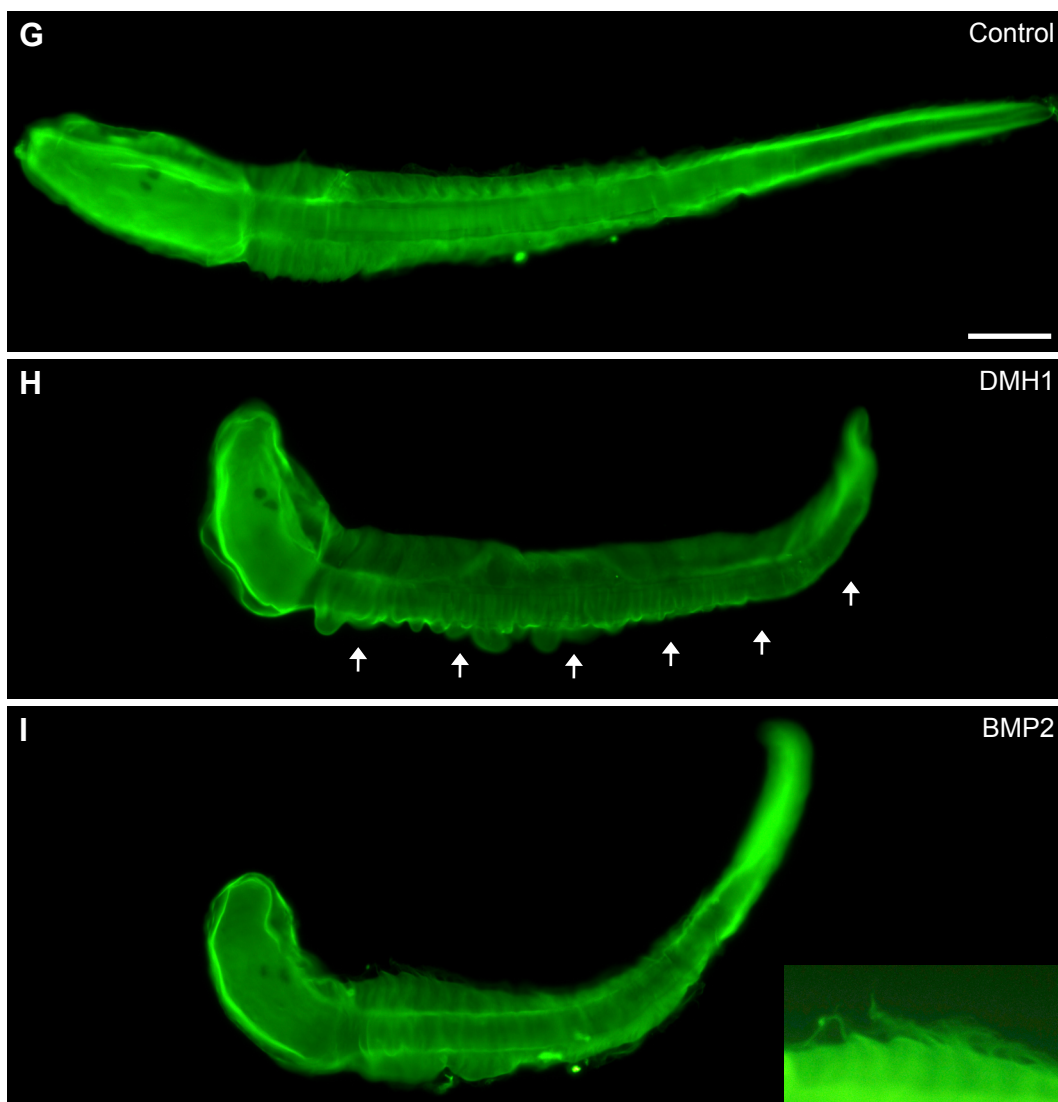

Supplement: Supplementary file 8 — Additional file 8: Fig. S3. Effects of BMP pathway modulation on larval tunic formation. (A-F) Phallusia mammillata. Overlay pictures between CBM3a-GFP and transmitted light for larvae from similar experiments as the ones described in Fig. 2 in lateral view (A,C,E) and dorsal views (B,D,F). (G-I) Ciona intestinalis. Dechorionated embryos were treated with DMSO and BSA (control, G, n=57), 2.5 µM DMH1 (H), or 150 ng/ml recombinant BMP2 protein (I) from early gastrula stages (St. 10). The resulting larvae were stained with CBM3a-GFP (green). Note the absence of ventral fin in DMH1-treated larva (white arrows; observed in all of the 87 larvae examined). The median fin of BMP2-treated larva appeared normal, but numerous fibers protruding outside the tunic were visible (inset in I, observed in 96% of the 55 larvae examined). Results from a single experiment. Scale bar: 100 µm. Individual data values can be found in Additional file 7: Table S1. [file 12915_2024_1872_MOESM8_ESM.pdf]

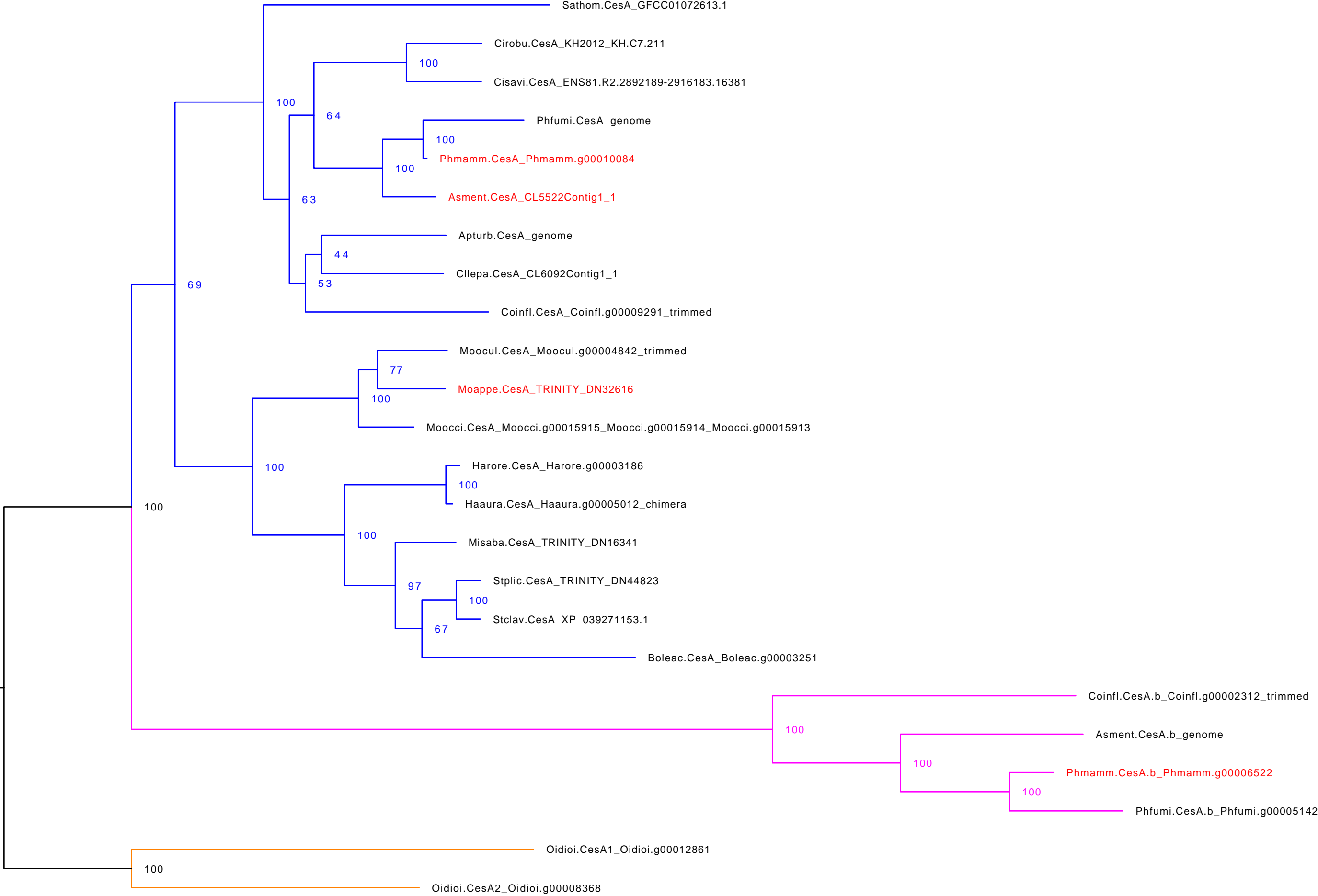

0.3

Supplement: Supplementary file 11 — Additional file 11: Fig. S5. Phylogenetic tree of CesA proteins in selected tunicate species. The tree was calculated using maximum likelihood (ML) method with IQ-TREE, and bootstrap supports are given at each node. Different groups are highlighted with colors: CesA (blue), CesA.b (purple), and CesA from Oikopleura (orange). The genes/proteins that are presented in this study are shown in red. [file 12915_2024_1872_MOESM11_ESM.pdf]

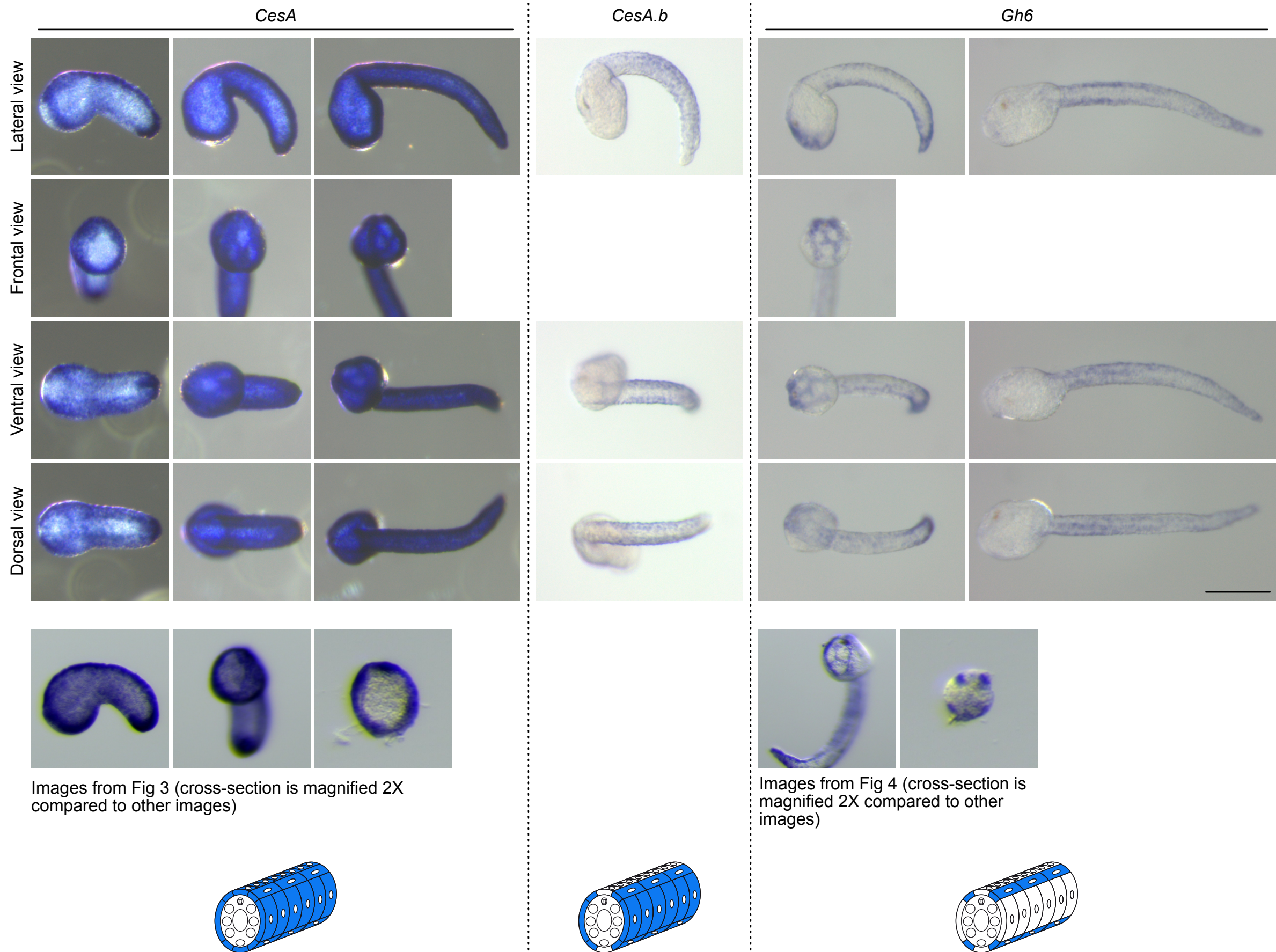

Supplement: Supplementary file 12 — Additional file 12: Fig. S6. Additional images of in situ hybridization for CesA, CesA.b and Gh6 in Phallusia mammillata. In the top part of the figure, for each gene, the same embryo has been imaged through different orientations. In the bottom part of the figure, some pictures already presented in Figs. 3 and 4 are shown for clarity, in particular cross-sections through the tail are twice bigger than other pictures. Schematic expression domains are highlighted in blue. Note that these schemes do not account for the tail tip region where medio-lateral rows of cells most likely are missing. In the tail tip, we suspect that CesA.b is expressed in the lateral cells and Gh6 in the median cells. Scale bar: 100 µm (except cross-sections: 50 µm). [file 12915_2024_1872_MOESM12_ESM.pdf]

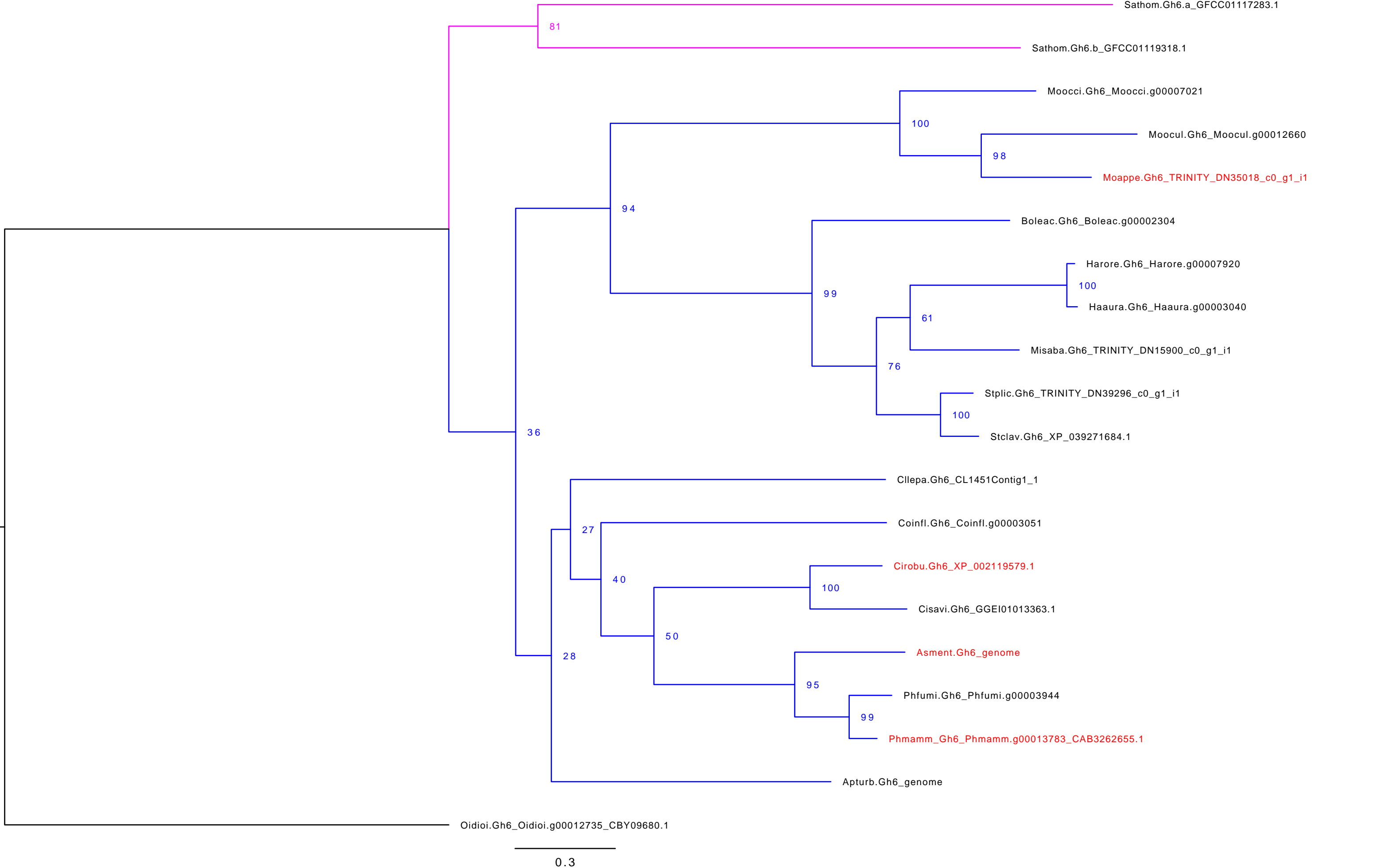

Supplement: Supplementary file 13 — Additional file 13: Fig. S7. Phylogenetic tree of Gh6 proteins in selected tunicate species. The tree was calculated using maximum likelihood (ML) method with IQ-TREE, and bootstrap supports are given at each node. Different taxonomic groups are highlighted with colors: ascidians (blue), thaliaceans (purple), and appendicularians (black). The genes/proteins that are presented in this study are shown in red. [file 12915_2024_1872_MOESM13_ESM.pdf]

*Phmamm.CesA*

*Phmamm.CesA.b*

*Phmamm.Gh6*

Control

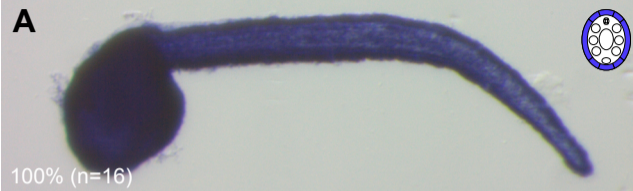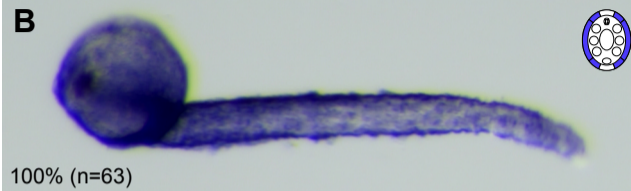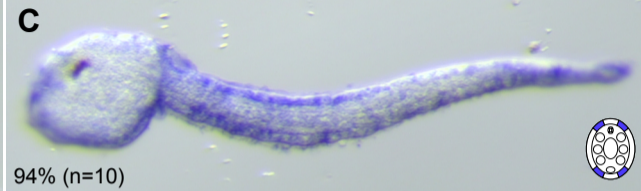

DMH1

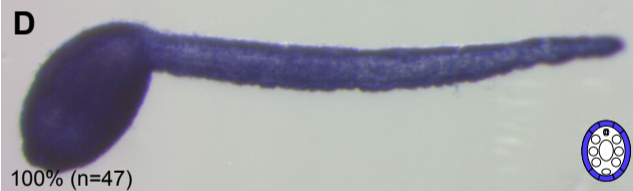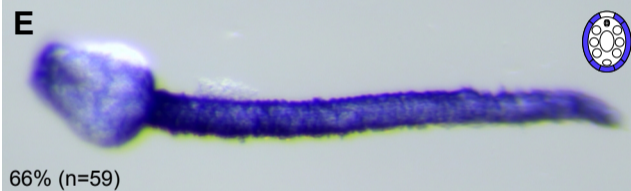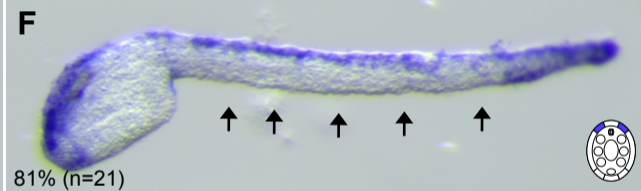

BMP2

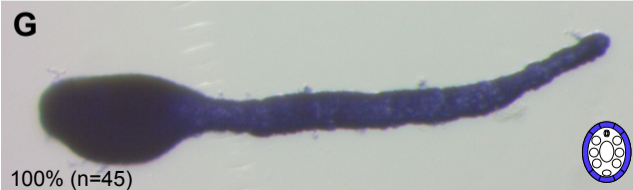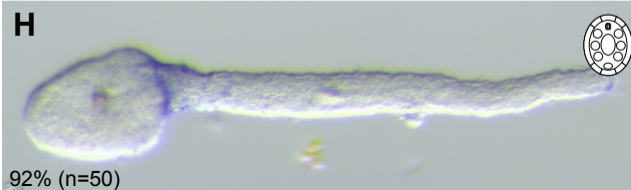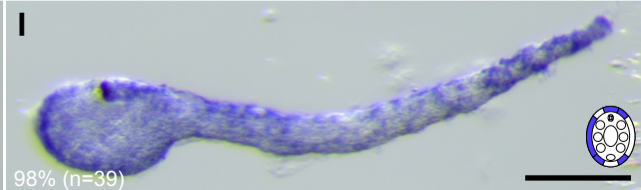

Supplement: Supplementary file 14 — Additional file 14: Fig. S8. HGT gene expression regulation by BMP signaling pathway in Phallusia mammillata. In situ hybridization at late tailbud stages (St. 24/25) for CesA (A,D,G), CesA.b (B,E,H) and Gh6 (C,F,I) in control (A-C), DMH1-treated (D-F) and BMP2-treated (G-I) embryos. CesA expression was expressed in the entire epidermis in all conditions. DMH1 treatment led to a loss of Gh6 expression in the ventral epidermis medio-lateral rows of cells (black arrows), and to an increased expression of CesA.b in the ventral tail epidermis midline. BMP2 treatment led to disorganized 'salt-and-pepper' pattern for Gh6 and to a loss of CesA.b expression. The results come from two experiments for CesA and Gh6, and three experiments for CesA.b (the averaged fraction of embryos displaying the phenotype and the number of embryos is shown on each panel). Individual data values can be found in Additional file 7: Table S1. Embryos are shown in lateral views with dorsal to the top and anterior to the left except for CesA.b that are ventral views with anterior to the left. For each image, a schematic cross-section through the tail depicts our interpretation of the patterns. Scale bar: 100 µm. [file 12915_2024_1872_MOESM14_ESM.pdf]

## Median fin

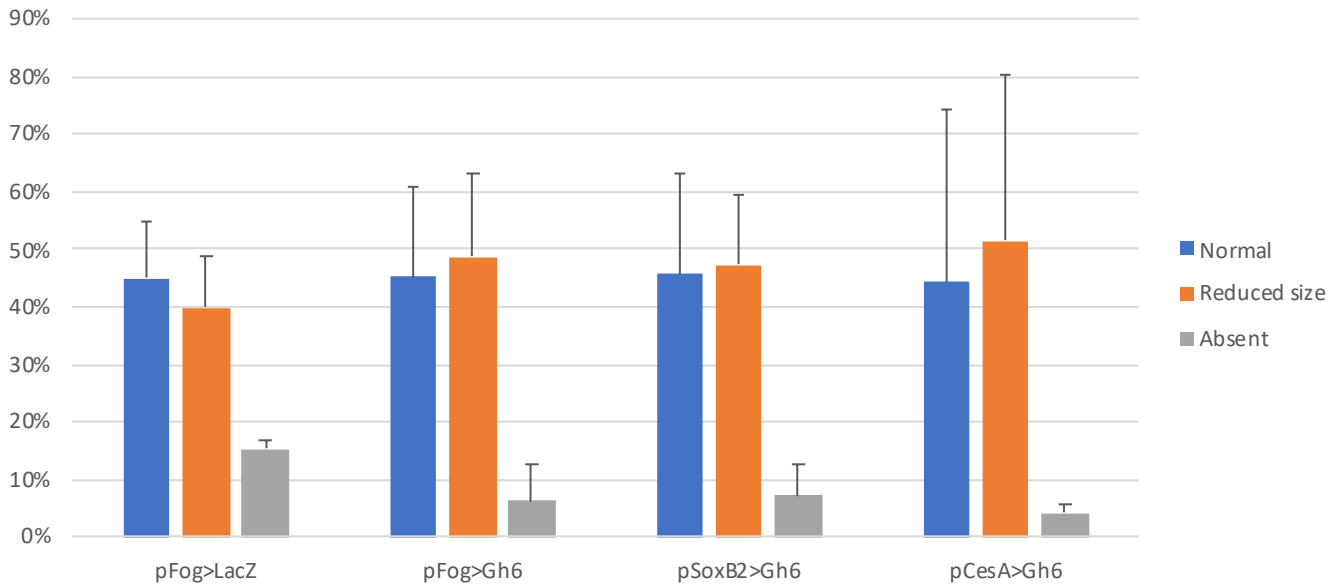

## Caudal fin

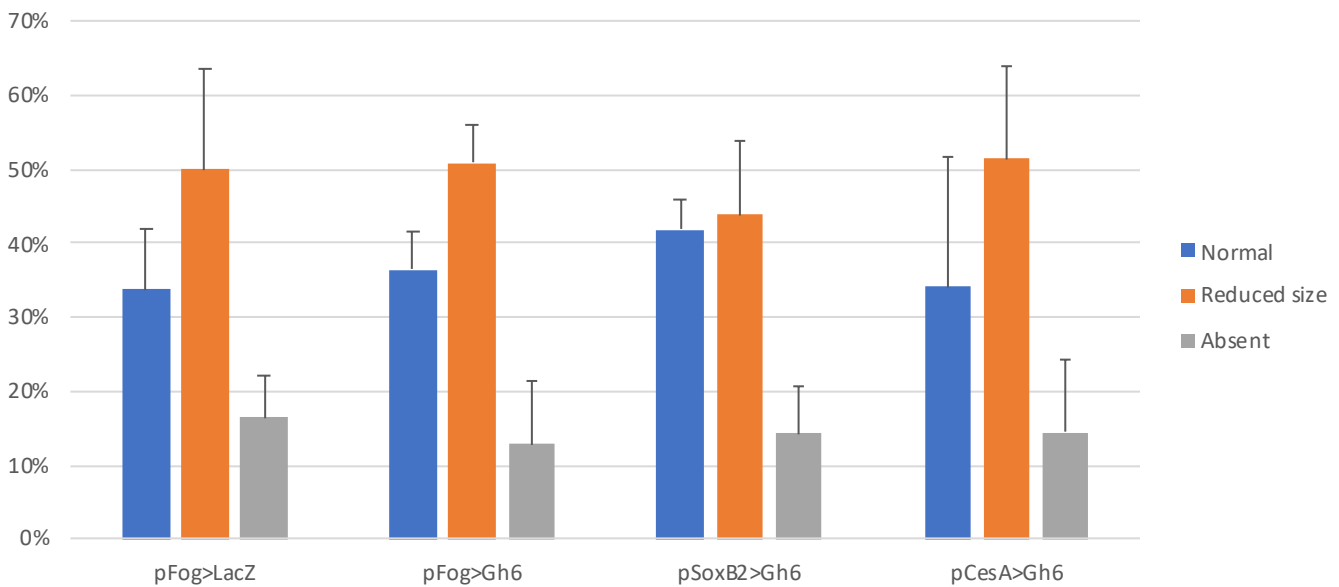

Supplement: Supplementary file 15 — Additional file 15: Fig. S9. Gh6 overexpression does not affect larval fin formation. Graphs representing scoring of median fin (top) and caudal fin (bottom) formation (following CBM3a-GFP staining) in larvae electroporated with the construct indicated on the graphs. The results come from 3 independent experiments with the following number of larvae examined: pFog>LacZ (234), pFog>Gh6 (148), pSoxB2>Gh6 (228), and pCesA>Gh6 (121). The error bars represent standard deviations. Individual data values can be found in Additional file 7: Table S1. [file 12915_2024_1872_MOESM15_ESM.pdf]
