# Supplementary material for: Molecular control of cellulosic fin morphogenesis in ascidians
Source: BMC Biol. 2024 Apr 2;22:74. doi: 10.1186/s12915-024-01872-7 (PMC10986139; doi:10.1186/s12915-024-01872-7)
Supplement: Supplementary file 2 — Additional file 2: Fig. S2. Tunic staining using DirecRed23 and Calcofluor White. Both dyes stained the embryos quite strongly, but they were useful in delineating the tunic. (Top panel) DirectRed23 and DAPI staining. The first picture shows an overlay of DirectRed23 and DAPI, and is not informative. However, masking the embryo's shape using the DAPI channel (thanks to the background staining of the entire cell and not only the nucleus) allows a clear visualization of the tunic. Results on the presence of the tunic before hatching are similar to the ones obtained with Calcofluor White (Fig. 1 and below). (Bottom panel) Calcofluor White and Sytox Green staining. Similar results were obtained using a similar approach with the confocal acquisitions shown in Fig. 1A-C. All images are maximum intensity projection from confocal z-stacks. [file 12915_2024_1872_MOESM2_ESM.pdf]

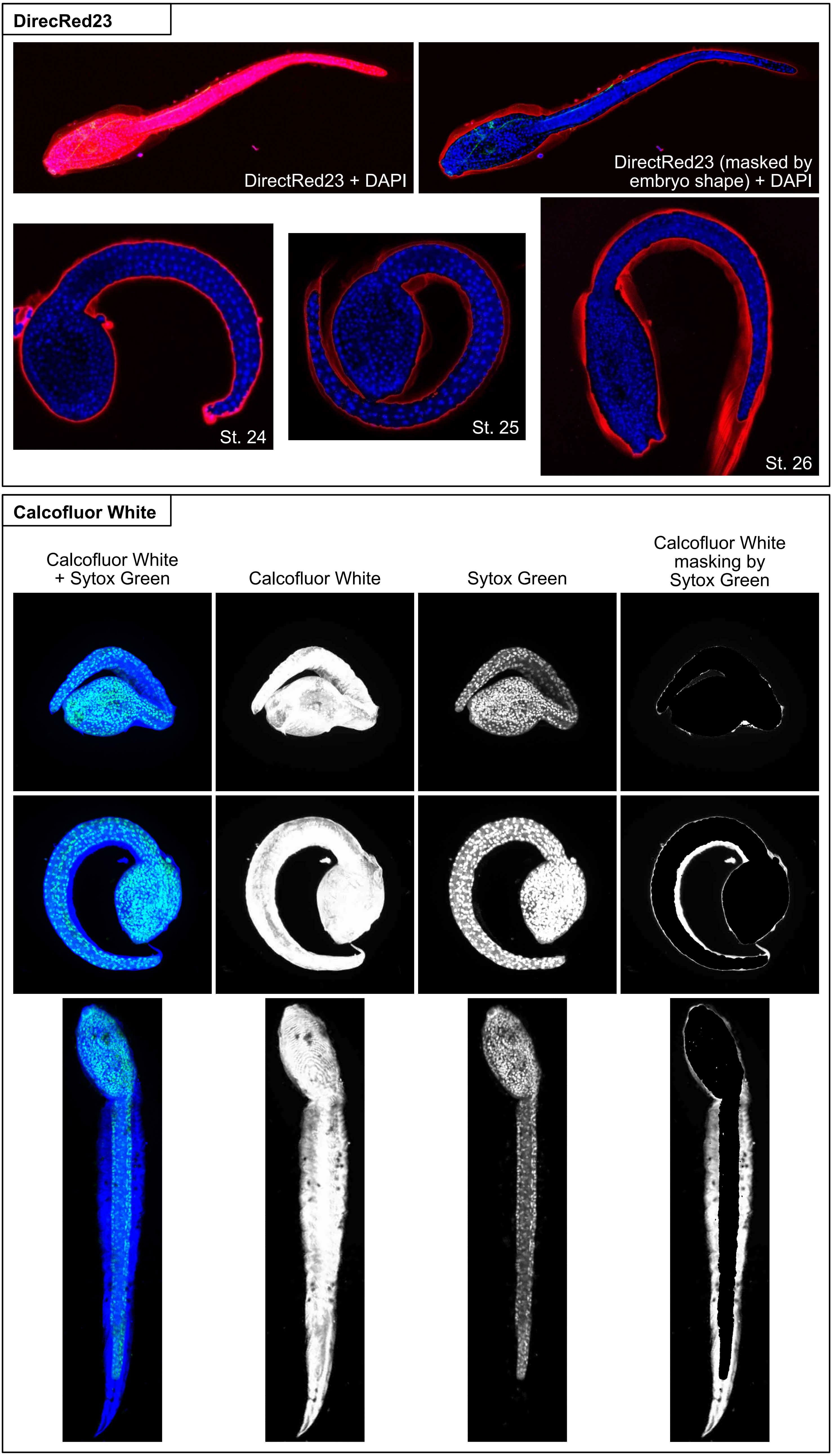
DirecRed23
DirectRed23 + DAPI
DirectRed23 (masked by embryo shape) + DAPI
St. 24
St. 25
St. 26
Calcofluor White
Calcofluor White + Sytox Green
Calcofluor White
Sytox Green
Calcofluor White masking by Sytox Green
