# Supplementary material for: Molecular control of cellulosic fin morphogenesis in ascidians
Source: BMC Biol. 2024 Apr 2;22:74. doi: 10.1186/s12915-024-01872-7 (PMC10986139; doi:10.1186/s12915-024-01872-7)
Supplement: Supplementary file 9 — Additional file 9: File S1. Evaluation of the mutagenesis triggered by CRISPR/Cas9. For each gene, a figure depicts: (top panel) the locus with the gene structure, essential protein domains, and the positions of sgRNA targets and PCR primers; (middle panel) a picture of an agarose gel for the different amplicons; and (bottom panel) results of Sanger sequencing at sgRNA targets' positions. Larvae with mutated locus are highlighted in orange. Each of these figures corresponds to a single experiment. [file 12915_2024_1872_MOESM9_ESM.pdf]

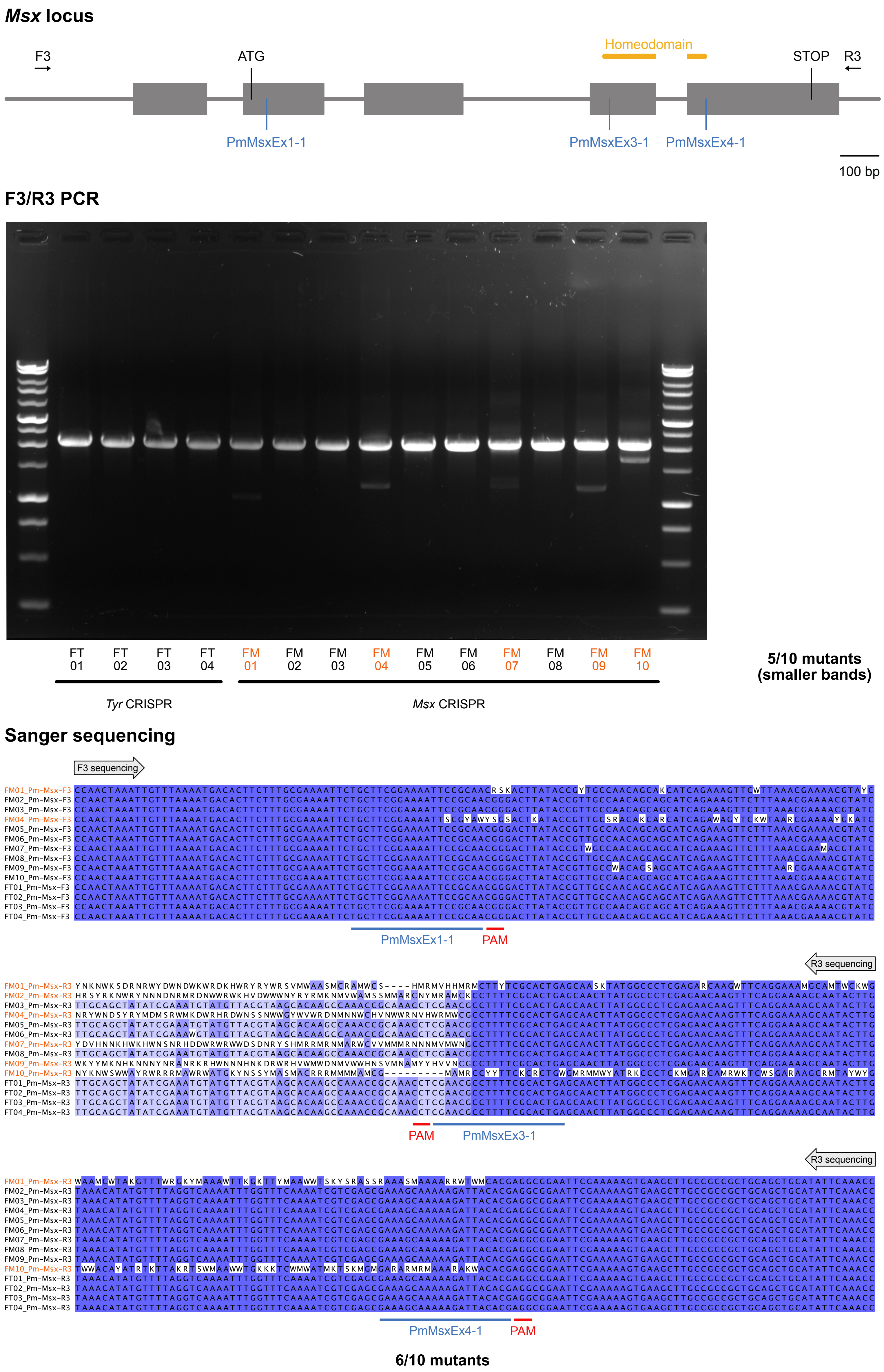
Msx locus
F3
ATG
Homeodomain
STOP
R3
PmMsxEx1-1
PmMsxEx3-1
PmMsxEx4-1
100 bp
F3/R3 PCR
FT 01
FT 02
FT 03
FT 04
FM 01
FM 02
FM 03
FM 04
FM 05
FM 06
FM 07
FM 08
FM 09
FM 10
5/10 mutants (smaller bands)
Tyr CRISPR
Msx CRISPR
Sanger sequencing
F3 sequencing
PmMsxEx1-1
PAM
R3 sequencing
PAM
PmMsxEx3-1
R3 sequencing
PmMsxEx4-1
PAM
6/10 mutants

Klf1/2/4/17 locus

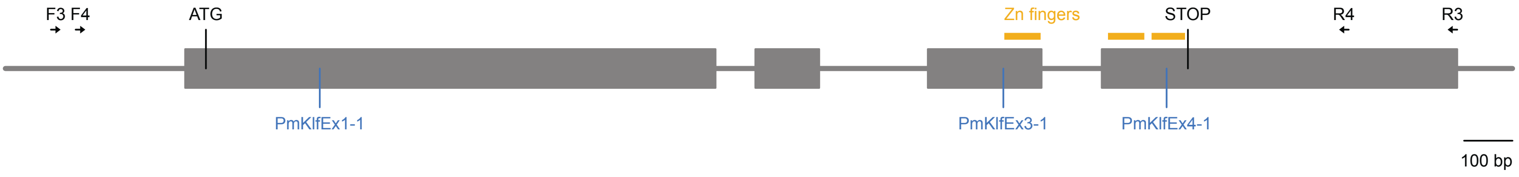

PCR2: F4/R4

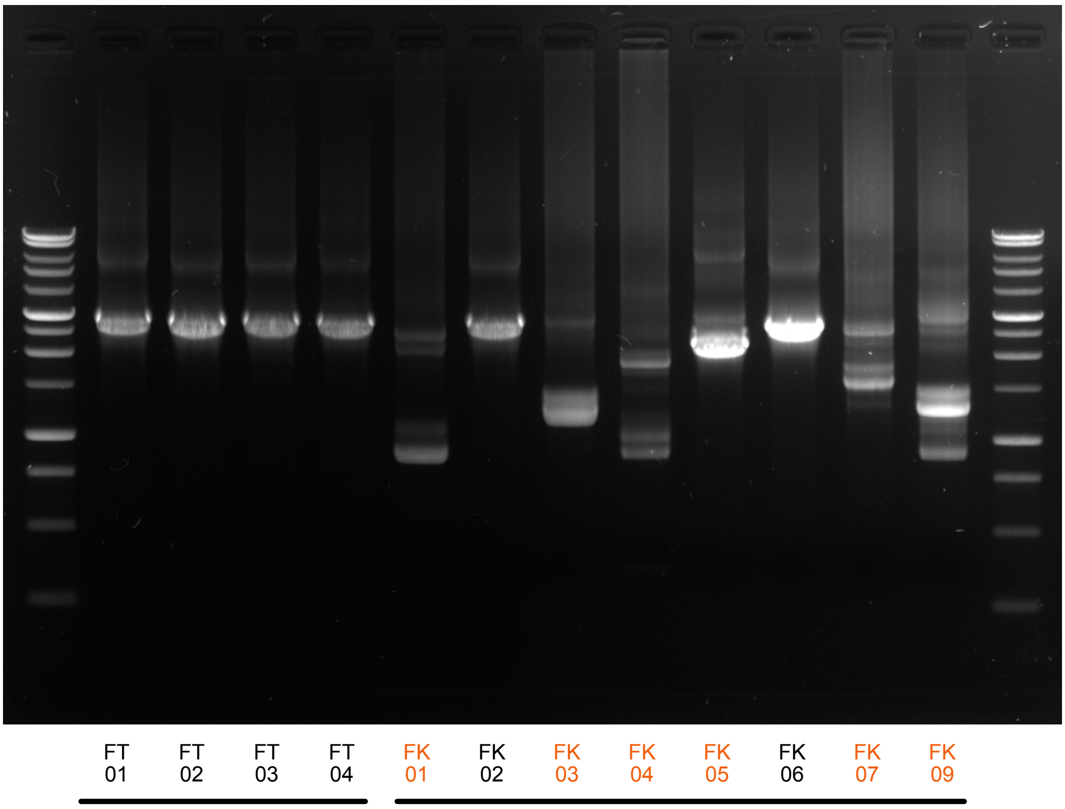

6/8 mutants (smaller bands)

Sanger sequencing

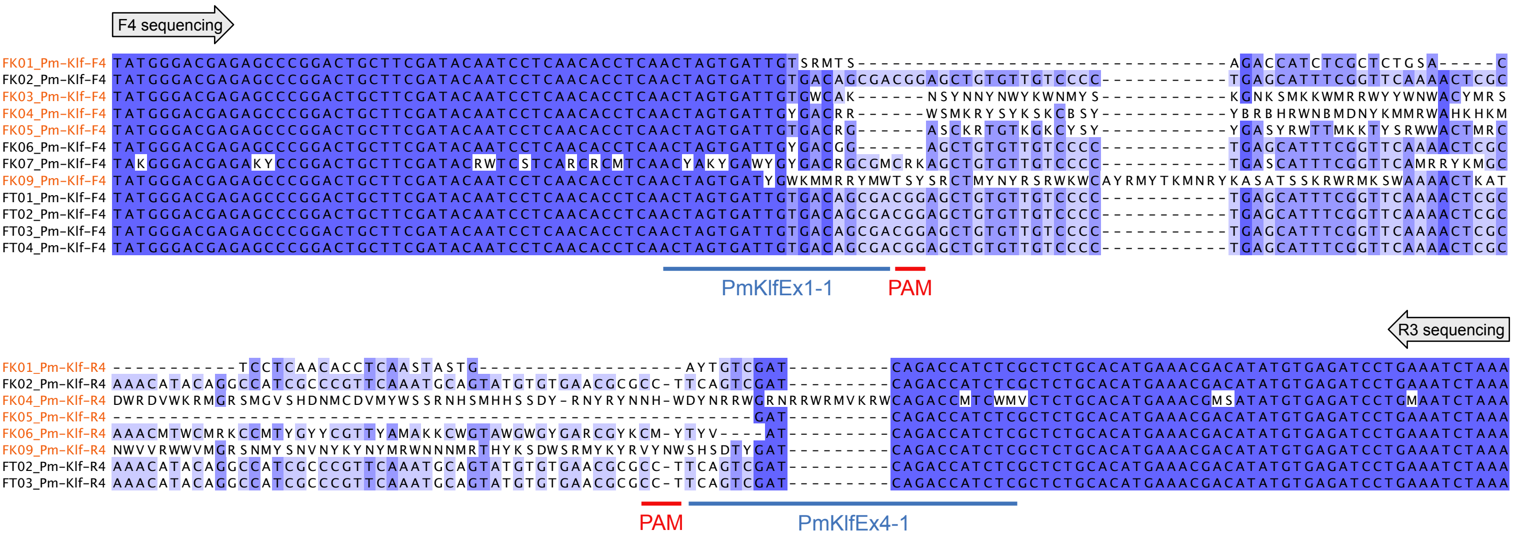

PmKlfEx3-1/PmKlfEx4-1 deletion

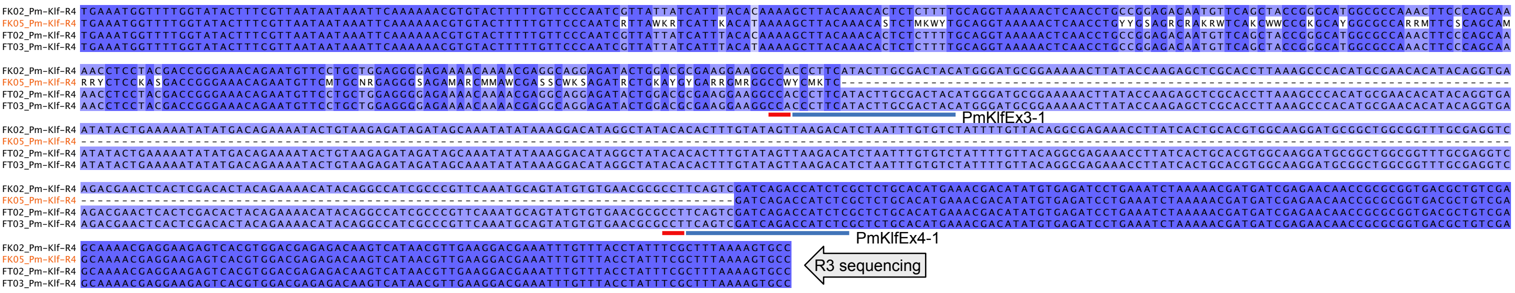

PmKlfEx1-1/PmKlfEx4-1 deletion

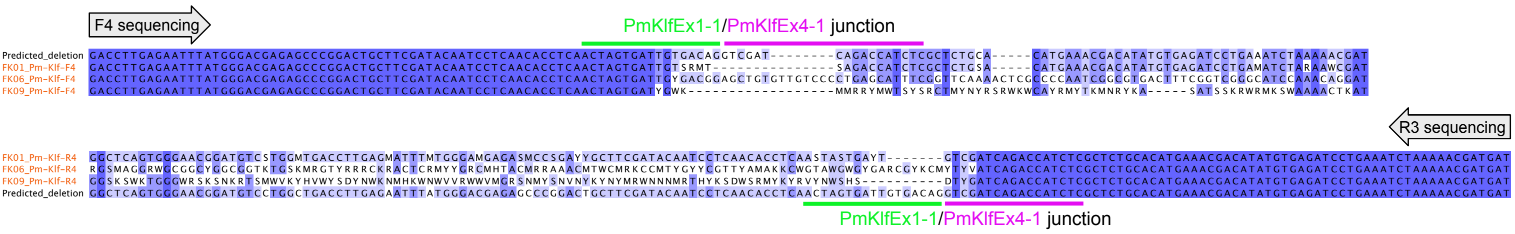

7/8 mutants

Gh6 locus

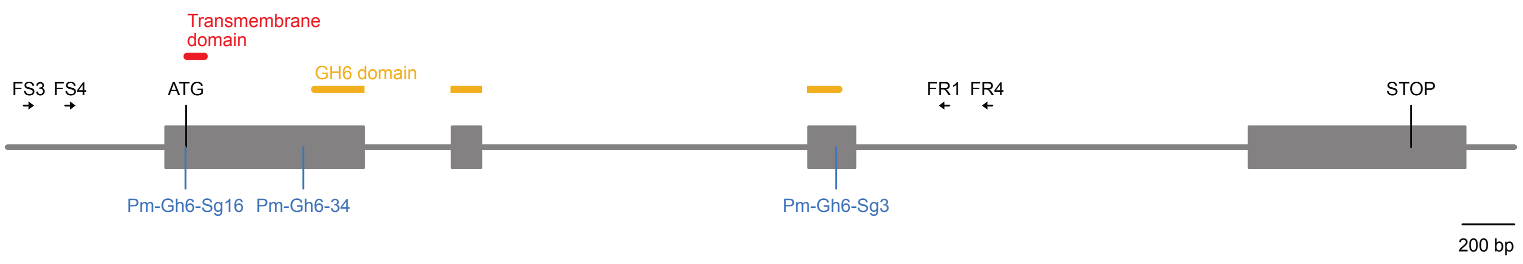

PCR2: FS4/FR1

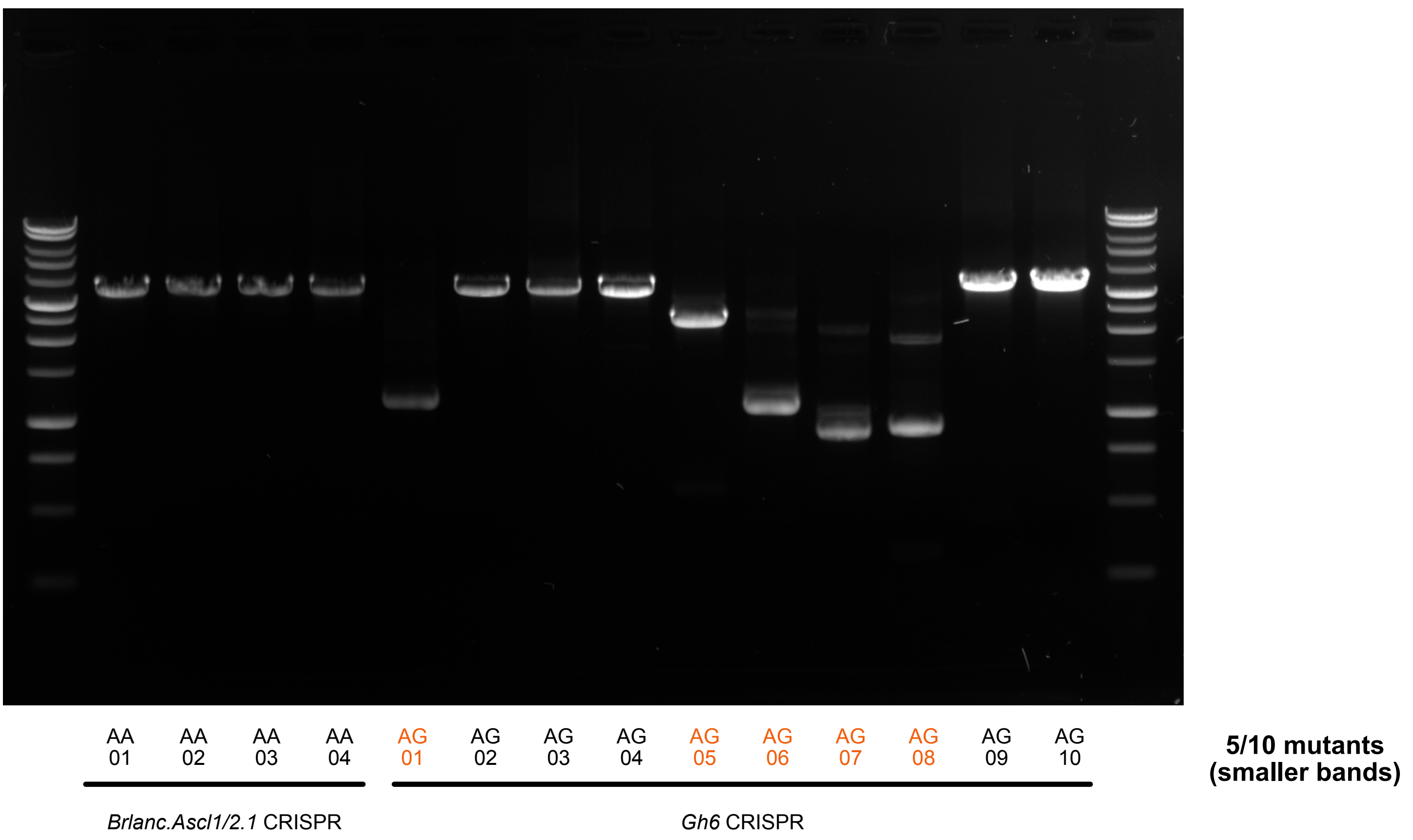

Sanger sequencing

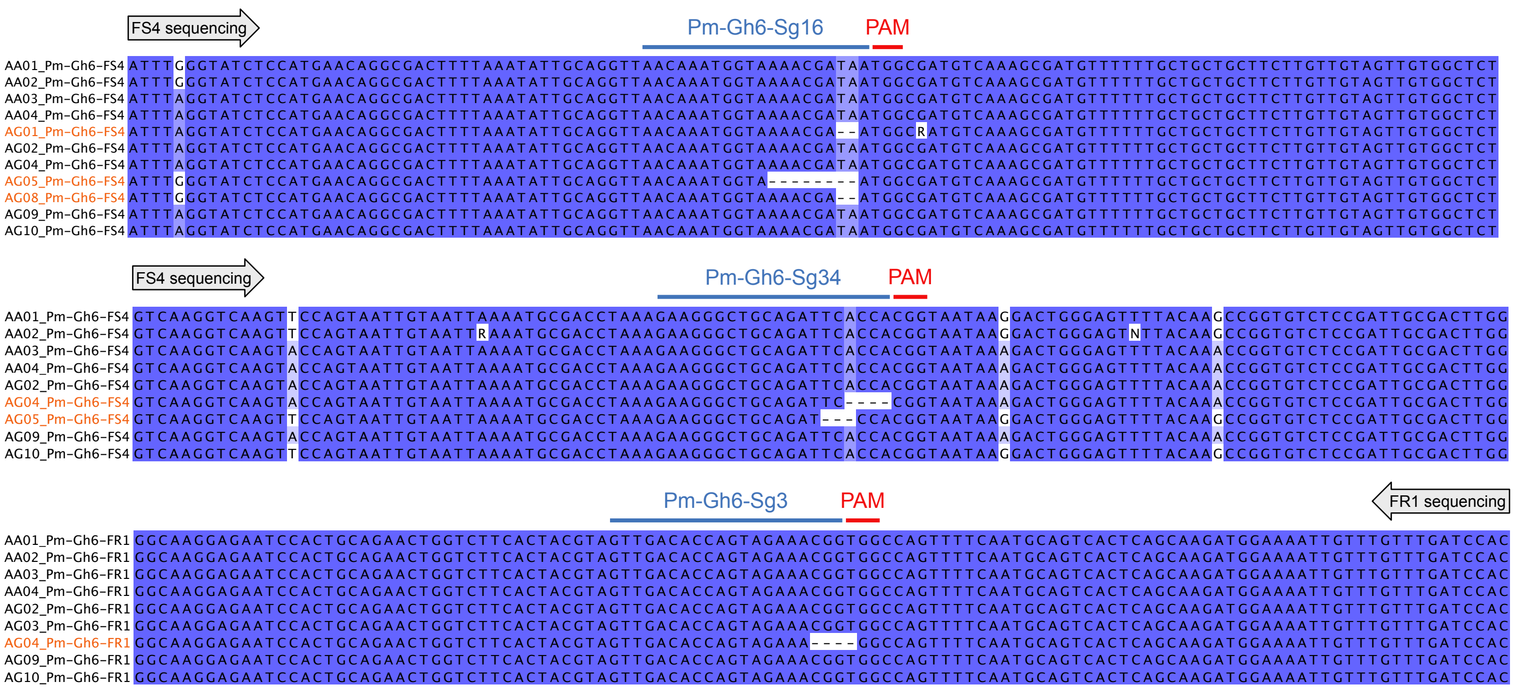

Pm-Gh6-Sg16/Pm-Gh6-Sg3 imperfect deletion — Pm-Gh6-Sg16/Pm-Gh6-Sg3 junction

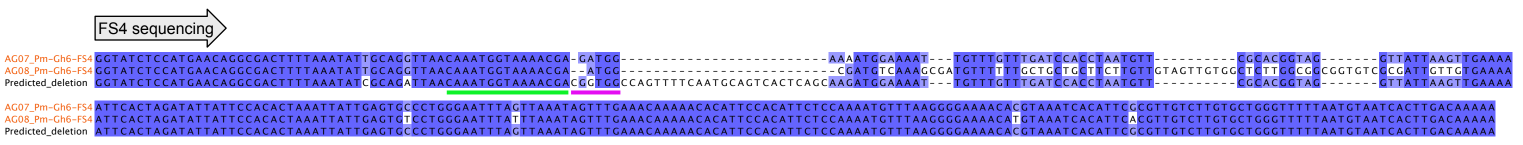

Large deletions

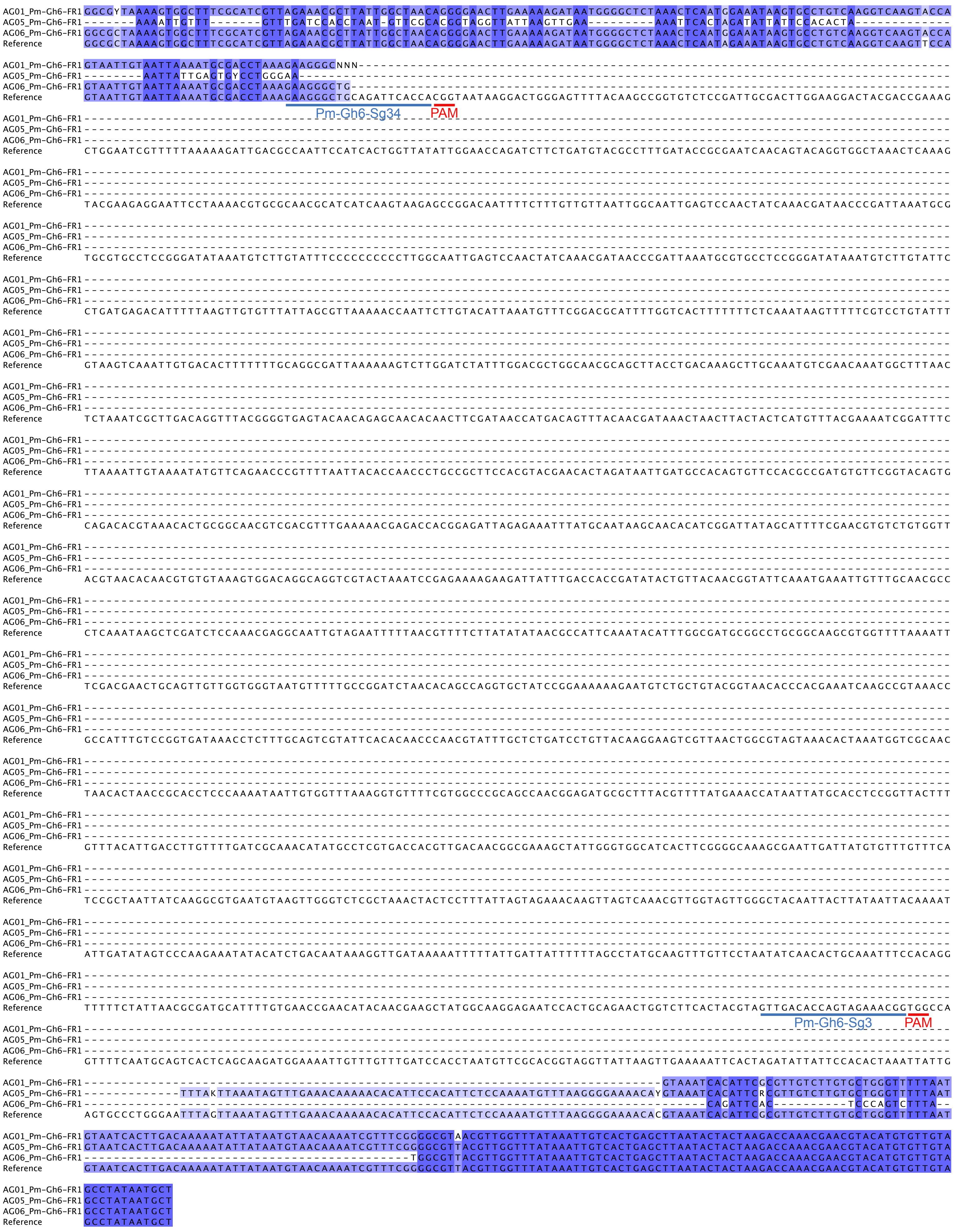

7/10 mutants
