# Supplementary material for: Molecular control of cellulosic fin morphogenesis in ascidians
Source: BMC Biol. 2024 Apr 2;22:74. doi: 10.1186/s12915-024-01872-7 (PMC10986139; doi:10.1186/s12915-024-01872-7)

CesA

```
>Phmamm.CesA_Phmamm.g00010084
MSHSGRDHELPGAYKRSNAGRNDSRSLPRSSFPPKPSRYTSSSFATSDDFGPSHFNFDDSDTSGSRSRNVSTLPSSAYPRHTSGSSFTSSQDPPPIP
DGKRRTISMGANHHPPGRQKSRERRRTMDARKHAPPFTISDSTRRTTSGSDDQHRLGSDGSSVYSSADSFDSQDDFDGRFPPRPPKPEKYLKRDTSS
SNFTGQFYPDPLESADMSRLQSGYSEDTNTTLTNGDRRPTDIDELAEYFNKKLEDVQRQADHAINEIQDGQRNRSFTDDSSMYSYNRGPPGGGTFAR
QGREPSTRTIRPSNRAGAGISSDASTITPTNSRSFRPDDQQSDNISAVRDELDSLKFQLSKGLENLENRGGSSQNMEEMADYFSKRLEECQKQVYSA
VNQAQNQPGETKQRFEGQPPSGRNRRSQDWGPAEEEFYDSSDSGIGPRNLTIRPMTRAHAEAQNNASVMPSAPPPVNRRQRHHEPERHDYPHPPPPM
DDEESFARMESMIPEKHHQAPPPIYRDDIPGGKMMDEDLLGSDDEEYWDDFEEFIDDDTPAPETSIPVPKITFKRHNYIYQVLYVVYILSFLAYVYV
RIQFTLDAPGLNRIYCIVVAILEIITAPSLILQGLCLWRWISRDPAPSDAFENPKFHTARIMVPTYKEPLEVVAGTVHEIIHMDLPPNFHVHVYVLD
DGKREILENWVLSKRTKRRVYLHYVARPKLPGVPHHAKAGNINHTLHYIFDDAYPEQECVIIFDADFMPRRNYLRRVLPVFSEKRTRPLALVQTPQF
FYNVNPEEDVWDHLNVSFFHRIEPSLDRWSAVNCCGTNFVVRADALKDVGYFPVGCLTEDTLLSLRLCTMGWGVAYHHEVLAIGQSPHEITEIFKQR
SRWCKGNLQIFLDEFPLMQSGLSLSQRIFYSSCGFNYFCASISIPFFQLVPAWAIFFGLWPVSEIGLEFAFAFFIYYLLGNMLLLFPPPGFGIRDMW
NGELASTNLWFTYFNGVRRIVGTKLLKGKGELTFKTTKKKTDEDEEGSNVGFQKEDVRACYMHFIMFFIMLITIIYAIVQASIAQNVQFYFYYMYMI
GMSWALINMTPYLIVVIYCWYRVRIPGLLVACFRNVQLMLRLVCAALIMVQAFTTRNLTENFTCPHEYTGVLGGSPLAMVKTTDALSKLQVARNSFW
LLGLDDDFYTMQQIKRTACEEDEVPIIVFFMHPSQGLALDSEAGHYMPEHRIDTWEDYDEKMSKYADHLSDIPSLIVMEPSLLMHTFNSKTEYHNTD
YQLAFSQRVGNVVKMFPYSWVYVDAGNAMYLQWQVNLDHIIGVMKNMPPAIRGFSINVGSFVNSTFNQQLAEEIHCQTGYHYIIDTSRNGGTFSDRS
LEEINQCTYDPPNAINGSKPMWMEGSKTTNIKSGTSQNTDLTVDSATEATTLRRKRWVLNQANTGNEPVLGEGPAAGAVYTSDTEYVAETANTGTDY
YDAYSDTASTLCISLAKVGLDAYAWVKTPGESDGRMFPAGTYHPCLMNHFSECSETCPQYVPKISGEFQRAESCSCS
```

GT2/Cellulose synthase
GH6

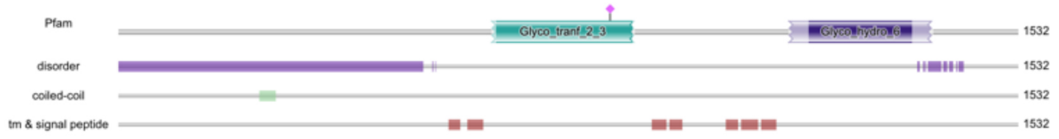

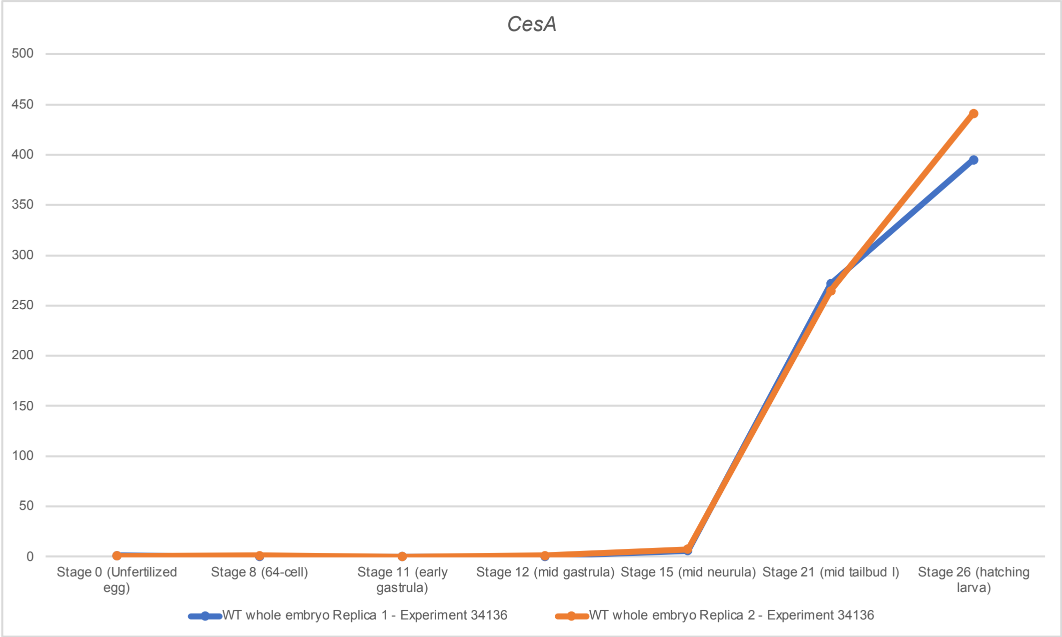

CesA.b

```
>Phmamm.CesA.b_Phmamm.g00006522
MFGDEDTASEPDIRMTELSDSDDETPIREASYPVPQVAFRATNLIYRILYILYLASFAVYVYVRLSFTLDAPGVSRFYCTFVAALEIVTAPALMIHG
LCLWKWYSRKPPVIDEKEQSMFHTARILVPTFKEPLEVVTGTVREIINLEIPEGLHIHVYVLDDGARRELEQWVANRRTKRYVYLHYVARPKLAGSP
HHAKSGNINHLLHFLYDQDCTENECVVVFDADFMPKRHFLTKVLPVFSEDRGGKPIALVQTPQHFYNVNPDEDMWDHMNTSFFHRTELGLDQWGAVN
CCGTNFVIRSDALIEANFLPVGCLTEDTLLSLRLCELGWAVAYHHEILAVGQAPNEISSIMKQRARWCKGNIQIFLDEFPLFNRRLSLPQRIFYSSL
GFNYICIAISTPIFQLIPVWAILFGLWPISHVNLEFAITFLVYYSLSNVILLYPPNGFGIKDMWNGELASINFWLTFFHALRGVLAAKFLGGELTFE
TTKKKTEDETERTQVGFRIQDLKMCFMHIFMLLFKLGAVVYAITKAITAASITNYYTSVIGMSWALMGMVPYAGVVIYCYYRVRIPGIVLACLRNIQ
LVLRVICIGLILTQALITQKESIIYNCPHDYQAELGIHPLTVARITDKISGQEVQRDAFWILGVKDDLYTIEQIKKLACEEKTVPVIVFAMLPKEGL
VLNAEISHYLPENQIETWEEYDARMLIYAEELRDIPSLIIMEPSLLMHTFNYETKYWNWNYQFAFFERINIMVRVYKRSWVYVDAGNPLFLQWQVNM
DYMAGILEQLQTTVRGFSVNVGSYVNTSHTTDVADALYCRTGLHYVIDTSRNGGVFSQRSMDEINDCKYDPPQVQEGHLPAWREGSKQVKLKVYNRL
ADLLVSQKRGNSLRFRRRADPMGLVILRDQNATCEPSNGDLDGLDAYLWIKTPGESDGRLFDSGTLHSCLLNHYIECSMACPQYVPKISGKFRRRKS
CQCVDDEEADDDYIDLT
```

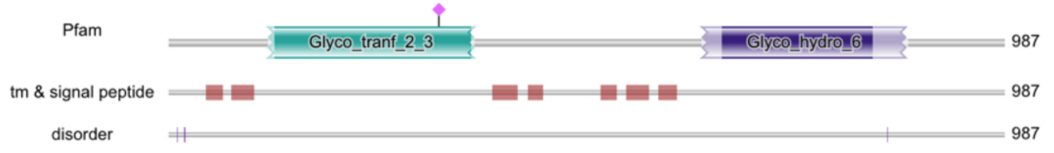

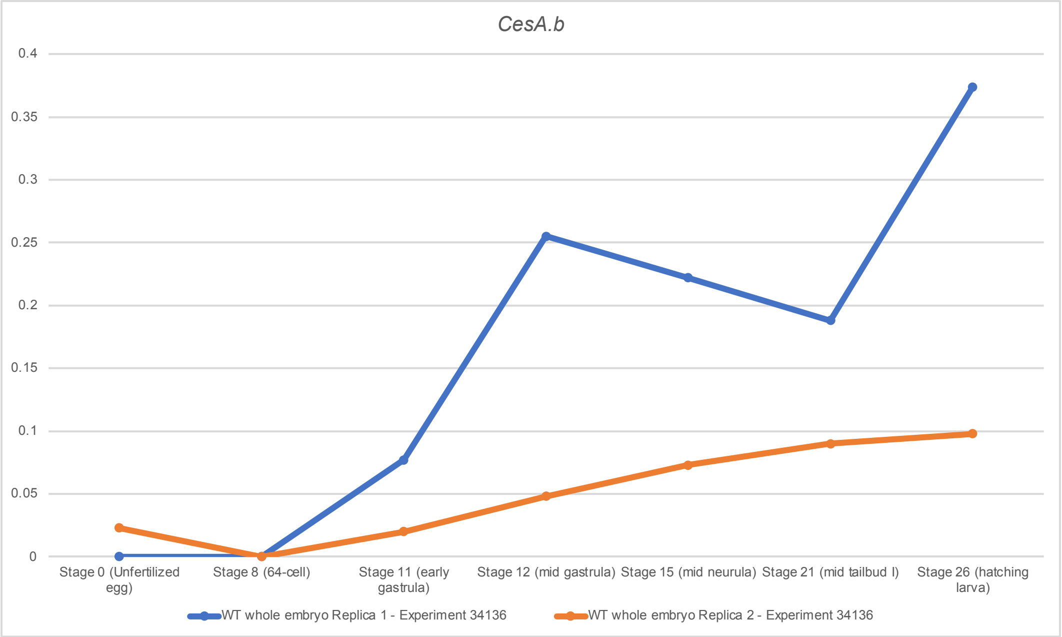

Supplement: Supplementary file 10 — Additional file 10: Fig. S4. Phmamm.CesA and Phmamm.CesA.b genes: predicted proteins and expression during embryogenesis. Identified protein domains using NCBI conserved domain and EMBL-EBI HMMER search engines [54, 55] are highlighted with colors. Expression levels across embryonic stages based on RNA-seq data were retrieved from the Aniseed database [56]. [file 12915_2024_1872_MOESM10_ESM.pdf]
